# *Leishmania donovani* resides in modified early endosomes by upregulating Rab5a expression via the downregulation of miR-494

**DOI:** 10.1371/journal.ppat.1006459

**Published:** 2017-06-26

**Authors:** Jitender Kumar Verma, Ruchir Rastogi, Amitabha Mukhopadhyay

**Affiliations:** National Institute of Immunology, New Delhi, India; Institut national de la recherche scientifique, CANADA

## Abstract

Several intracellular pathogens arrest the phagosome maturation in the host cells to avoid transport to lysosomes. In contrast, the *Leishmania* containing parasitophorous vacuole (PV) is shown to recruit lysosomal markers and thus *Leishmania* is postulated to be residing in the phagolysosomes in macrophages. Here, we report that *Leishmania donovani* specifically upregulates the expression of Rab5a by degrading c-Jun via their metalloprotease gp63 to downregulate the expression of miR-494 in THP-1 differentiated human macrophages. Our results also show that miR-494 negatively regulates the expression of Rab5a in cells. Subsequently, *L*. *donovani* recruits and retains Rab5a and EEA1 on PV to reside in early endosomes and inhibits transport to lysosomes in human macrophages. Similarly, we have also observed that *Leishmania* PV also recruits Rab5a by upregulating its expression in human PBMC differentiated macrophages. However, the parasite modulates the endosome by recruiting Lamp1 and inactive pro-CathepsinD on PV via the overexpression of Rab5a in infected cells. Furthermore, siRNA knockdown of Rab5a or overexpression of miR-494 in human macrophages significantly inhibits the survival of the parasites. These results provide the first mechanistic insights of parasite-mediated remodeling of endo-lysosomal trafficking to reside in a specialized early endocytic compartment.

## Author summary

*Leishmania donovani* causes visceral leishmaniasis in human. This parasite is thought to reside and replicate in a phagolysosomal compartment in macrophages. But, how *Leishmania* survives in such a detrimental compartment in macrophages is not known. In contrast, most of the intracellular pathogens avoid targeting to lysosomes in the host cells. We have found that *Leishmania* upregulates the expression of Rab5a, an early endosomal protein, by downregulating the expression of miR-494 in infected human macrophage as a consequence of gp63-dependent degradation of c-Jun. Subsequently, parasites recruit and retain Rab5a and EEA1 on their parasitophorous vacuoles demonstrating that *Leishmania* resides in the early endosomal compartment and consequently inhibits their transport to the lysosomes. However, we have found that parasites also recruit Lamp1 and inactive pro-CathepsinD on PV which led to previous conclusion that *Leishmania* resides in the phagolysosomal compartment. We have also shown that parasites block the processing of the lysosomal enzymes in the early endosomal compartment by overexpressing Rab5a which also helps the parasites to survive in human macrophages. Finally, we have shown that overexpression of Rab5a by downregulating miR-494 in macrophages is essential for parasite survival. These results unequivocally prove that *L*. *donovani* resides in a modified early compartment in human macrophages.

## Introduction

*Leishmania donovani* (Ld) is an obligate intracellular parasite which causes visceral leishmaniasis in the mammalian hosts that affects annually about 12 million people worldwide [1]. This parasite is postulated to reside and replicate in a phagolysosomal compartment in mouse macrophages as the parasites acquire lysosomal markers such as Lysosome Associated Membrane Protein 1 (Lamp1), Lamp2 and CathepsinD on PV [2,3]. However, not much is known about how *Leishmania* is surviving in such detrimental compartment. Interestingly, it has been shown that *Leishmania* excludes Vesicular Proton-ATPase on PV by inhibiting the recruitment of Synaptotagmin V to prevent the acidification in mouse macrophages [4]. Consequently, some recent studies have shown that *Leishmania* also modulates the recruitment of Rab7 [5] and ER markers [6] on PV in mouse macrophages. Thus, the *Leishmania* possibly resides in a hybrid compartment, but the nature of the compartment is not well characterized [7].

Since Rab GTPases are the central regulators of membrane trafficking pathways [8,9,10], targeting the function of Rab proteins is one of the commonly used mechanisms exploited by intracellular pathogens to subvert their lysosomal targeting [11,12,13,14,15,16]. Moreover, successful intracellular pathogens are also shown to modulate the expression of host cytokines to establish a safe niche inside the host cells [17,18,19]. Consequently, we have shown that cytokines can specifically modulate the expression of different Rabs [20]. Thus, it is possible that intracellular pathogens might also modulate the expression of Rab GTPases in the host cells. In addition, recent studies have shown that several intracellular pathogens such as *S*. *typhimurium*, *M*. *tuberculosis* and *L*. *monocytogenes* target the host microRNAs (miRNA) to modulate the expression of host proteins for their successful infection and survival [21,22,23]. Thus, it is tempting to speculate that *Leishmania* might target the host miRNA(s) to modulate the expression of endocytic Rab GTPases in infected macrophages.

Here, we have shown that *L*. *donovani* upregulates the expression of Rab5a in human macrophages by inhibiting the expression of miR-494 and retains Rab5a and EEA1 on PV to survive in an early endocytic (EE) compartment. Thus, in contrast to previous perception, our results demonstrate that *L*. *donovani* modulates endo-lysosomal pathway to reside in a modified early endocytic compartment and inhibits lysosomal transport in macrophages.

## Results

### *Leishmania* specifically upregulates the expression of Rab5a in macrophages

**Fig 1:**
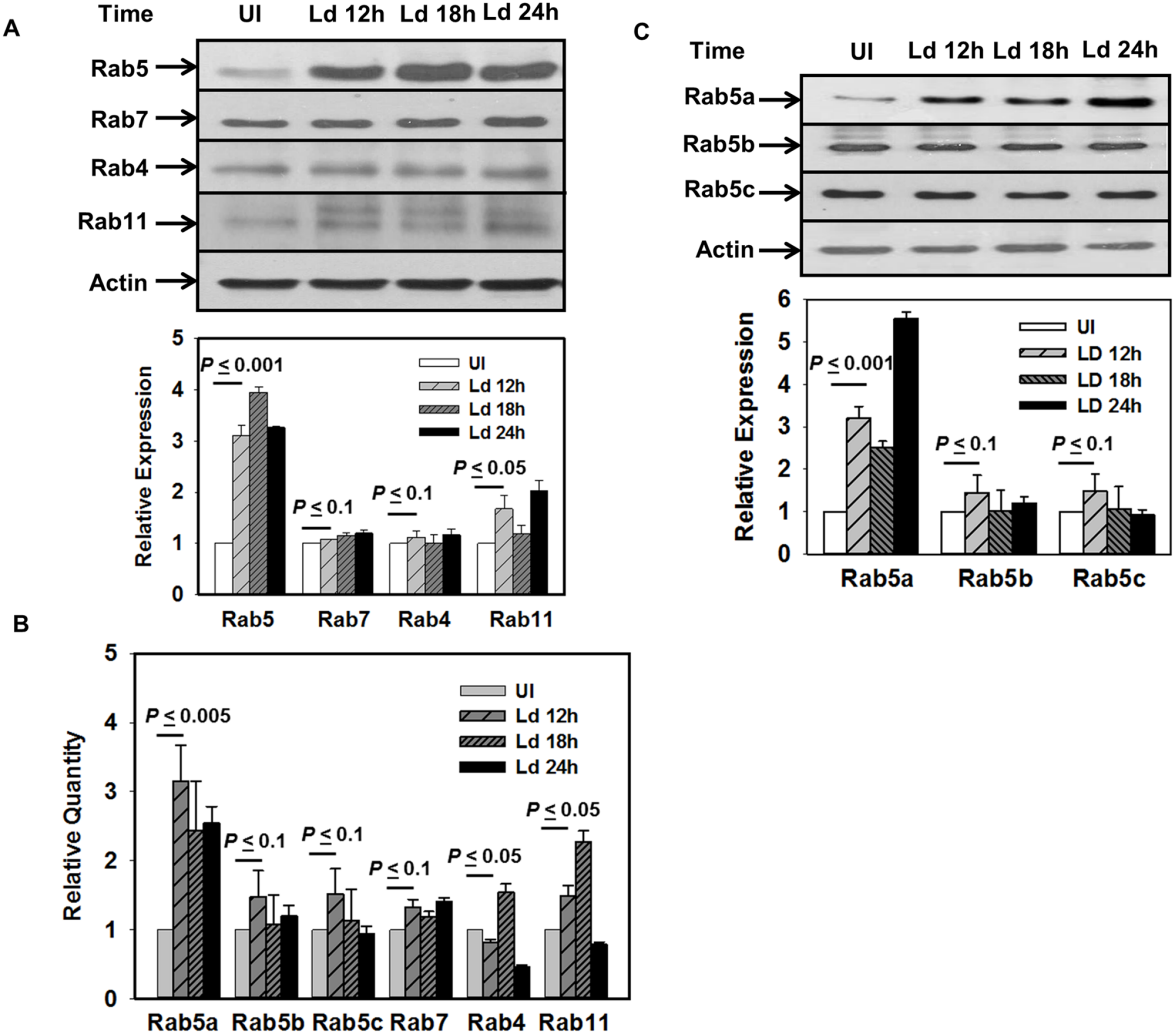
*Leishmania donovani* infection specifically upregulate the expression of Rab5a in macrophages. A. *Leishmania* infected THP-1 differentiated human macrophages were lysed at different time points after infection and levels of indicated Rabs were determined by Western blot analysis using specific antibodies. Actin was used as loading control. Lower panel indicates the quantitation of the respective data. Lower band of Rab11 corresponding to uninfected control was only used for quantitation of Rab11 in infected cells. B. Levels of different Rabs in infected and uninfected human macrophages at indicated time points were determined by qPCR as described in Materials and Methods. C. Levels of different isoforms of Rab5 in uninfected and infected human macrophages were determined by Western blot analysis using specific antibodies. All results are represented as mean ± S.D. of three independent experiments and normalized to respective control. Expression of normalized indicated Rab in uninfected cells was arbitrarily chosen as one unit. Results of the indicated groups were analyzed by paired *t* test and levels of significance are indicated by *P* value.

To determine whether *Leishmania* infection modulates the expression of different Rab GTPases, human THP-1 monocytic cell line were differentiated into macrophages (human macrophages) and were infected with *L*. *donovani* promastigotes (MOI 1:20). Subsequently, the cellular contents of various Rabs were determined by Western blot analysis using specific antibodies at indicated time points of infection. About 4-fold and 2-fold increase in the cellular content of Rab5 and Rab11, respectively, were observed after 24 h of infection (Fig 1A) in comparison to uninfected control cells. No significant change in the levels of expression of Rab4 and Rab7 were observed in infected and uninfected cells.

To determine whether higher level of Rab5 expression was due to transcriptional activation, we checked transcript levels of different endocytic Rab GTPases by Real Time PCR (qPCR) in infected human macrophages. Similar to protein expression, our results showed about 3-fold and 2-fold induction in the levels of Rab5a and Rab11 mRNA expression, respectively, after 24 h of *L*. *donovani* infection in comparison to uninfected control cells (Fig 1B). Previous studies demonstrated that Rab5 has three isoforms namely, Rab5a, Rab5b and Rab5c in mammalian cells [24]. Interestingly, we observed that *L*. *donovani* infection does not significantly alter the expression of Rab5b and Rab5c (Fig 1B). These results were further confirmed by Western blot analysis using isoform-specific antibodies (Fig 1C) and limited dilution semi-quantitative RT-PCR (S1 Fig).

### *Leishmania donovani* infection downregulates the expression of miR-494 by degrading c-Jun in macrophages

**Fig 2:**
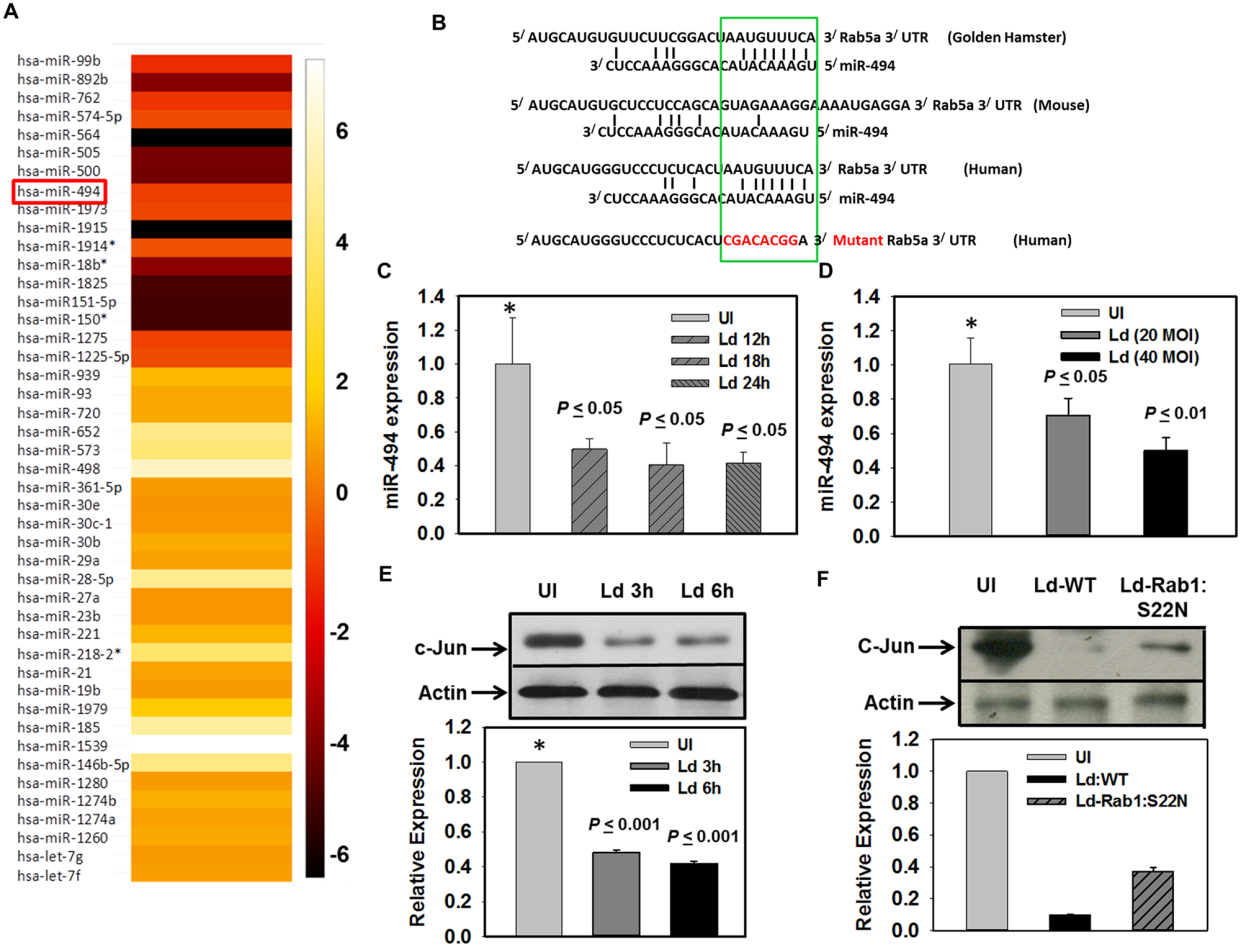
*Leishmania donovani* downregulates the expression of miR-494 by degrading c-Jun in macrophages. A. *Leishmania* infection modulates the expression of several miR in infected human macrophages as revealed by microarray analysis. Expression fold values are provided in terms of log base 2. Colour scale shown in the right illustrates the relative expression level of miRNAs in *Leishmania* infected macrophages in comparison to uninfected control. Yellow colour represents an expression level above the mean and Red colour represents the expression level lower than the mean. The whole microarray data have been submitted in Gene Expression Omnibus database (accession number GSE89529). B. TargetScan prediction algorithms showing that 3^/^-UTR of Rab5a of human and hamster contains an 8-mer target site that precisely matches the seed region of miR-494. C. THP-1 differentiated macrophages were infected with *Leishmania* (MOI 1:20) and level of expression of miR-494 in infected cells was determined at indicated time by qPCR as described in Materials and Methods. D. Similar experiments were carried out with indicated MOI of infection and level of miR-494 was detected after 12 h. E. THP-1 differentiated human macrophages were infected with *Leishmania* (MOI 1:20) and level of c-Jun in infected cells was determined at indicated time by Western blot analysis using specific antibody. Actin was used as control. F. Similar experiments were carried out with gp63 secretion deficient transgenic parasites (Ld-Rab1:S22N) and level of c-Jun in infected cells was determined after 6 h of infection by Western blot analysis using specific antibody. All results are represented as mean ± S.D. of three independent experiments and normalized to respective control. Expression of normalized miR-494 or c-Jun in uninfected cells in respective experiment was arbitrarily chosen as one unit. Results of uninfected (*) and infected cells were analyzed by paired *t* test and levels of significance are indicated by *P* value.

To understand the mechanism of upregulation of Rab5a expression in *Leishmania* infected human macrophages, we compared the miRNA profiles of uninfected and infected macrophages. Our results showed that expressions of 29 miRNAs are upregulated by more than 2-fold in *L*. *donovani* infected macrophages whereas expressions of 19 miRNAs are found to be downregulated by about 2-fold in infected cells in comparison to uninfected cells (Fig 2A). As miRNA negatively regulates the expression of their target gene, we screened those miRNAs which were downregulated in *Leishmania* infected human macrophages to identify their putative target sites in 3^/^-regulatory region of Rab5a using TargetScan prediction algorithms. This analysis predicted that 3^/^-UTR of Rab5a mRNA of human cells contains an 8-mer target site (5/-AUGUUUCA-3/) located between 191–198 nucleotides that precisely matches the seed region (positions 1–8) of miR-494 (Fig 2B). Interestingly, we observed that 8-mer target site of miR-494 is well conserved in the 3^/^-UTR of Rab5a in hamster whereas it is completely absent in the 3^/^-UTR of Rab5a in mice (Fig 2B). Our analysis also predicted (S2 Fig) that miR-494 has highest affinity to its complimentary target site for Rab5a (mirSVR score: -1.2494) compared to Rab5b (mirSVR score: -0.0086) and Rab5c (mirSVR score: -0.1579) in human cells.

Consequently, we determined the level of expression of miR-494 in *L*. *donovani* infected human macrophage (MOI 1:20) at different time periods of infection. Our results showed about 60% inhibition of the expression of miR-494 in *L*. *donovani* infected macrophages after 24 h of infection in comparison to uninfected cells (Fig 2C). We also found that infection with MOI (MOI 1:40) led to higher suppression of miR-494 than with MOI (MOI 1:20) after 12 h of infection (Fig 2D). These results indicated that inhibition of miR-494 expression in infected macrophages is also dependent on the extent of infection. To determine how *L*. *donovani* infection downregulates the expression of miR-494 in macrophages, we checked for the level of c-Jun subunit of AP-1 transcription factor in infected cells as it was previously reported that miR-494 expression is regulated by AP-1 transcription factor [25]. Previous studies demonstrated that AP-1 is composed of c-Jun, c-Fos family of proteins. Interestingly, we found that c-Jun subunit level is significantly lower (~50%) in human macrophages after 6 h of infection with *L*. *donovani* in comparison to uninfected cells (Fig 2E). It was reported earlier that *Leishmania* gp63 is a metalloprotease which is secreted into host cells and degrades several host proteins including some transcription factors like NF-kB, STAT1 and AP-1 to alter gene expression [26,27]. Therefore, to determine how *Leishmania* infection lowers the c-Jun level in infected macrophages, we infected the human macrophages with *L*. *donovani* overexpressing LdRab1:S22N, a gp63 secretion deficient parasite [28]. Interestingly, our results showed (Fig 2F) that degradation of c-Jun is significantly lower in LdRab1:S22N infected cells in comparison to infection with Ld:WT cells. These results were substantiated by the fact that overexpression of gp63 in Raw 264.7 mouse macrophages significantly degrades c-Jun (S3 Fig). Thus, *Leishmania* degrades c-Jun in infected human macrophages via their gp63 to inhibit the expression of miR-494.

### miRNA-494 regulates the expression of Rab5a

**Fig 3:**
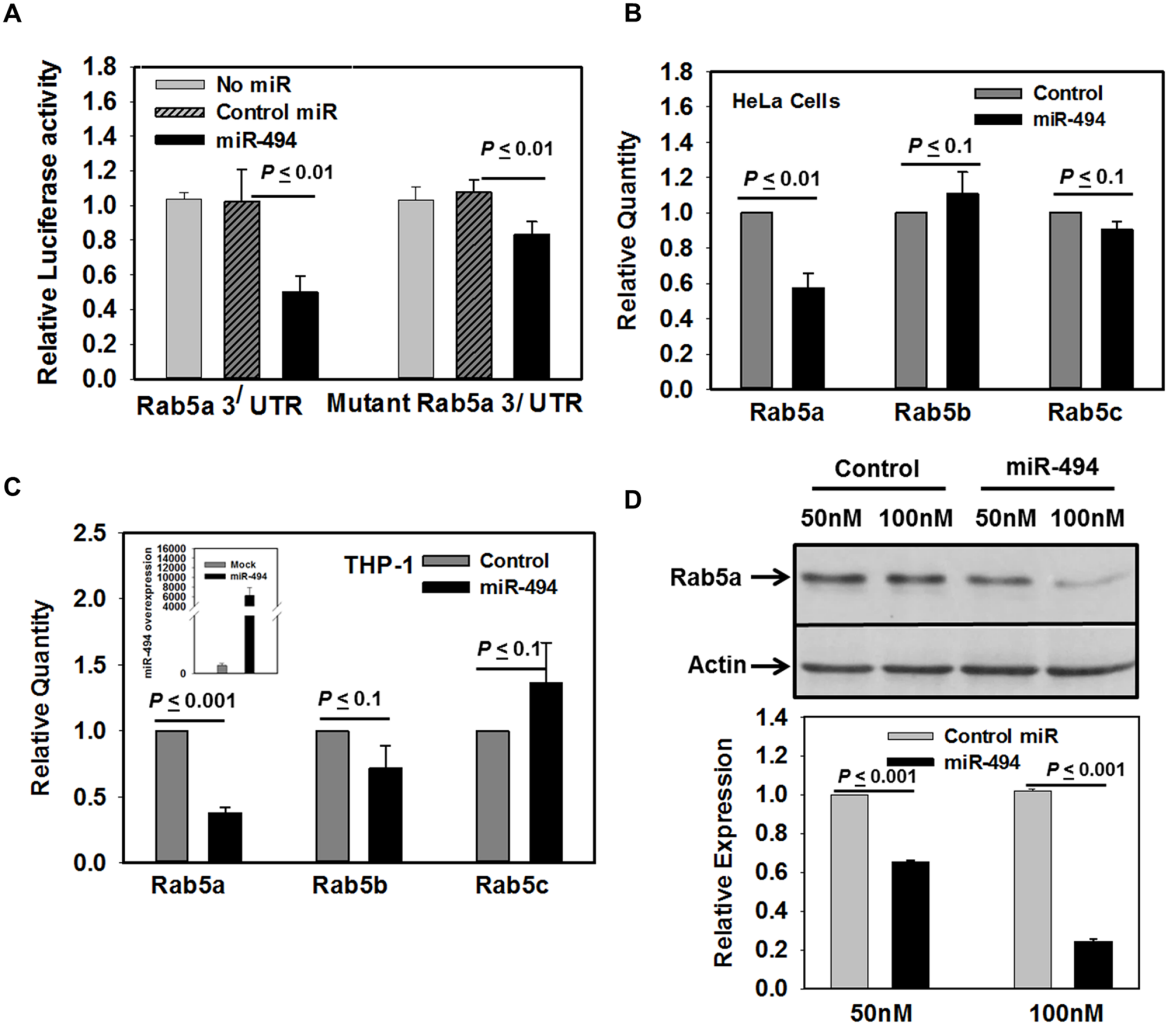
miR-494 negatively regulates the expression of Rab5a. A. To determine miR-494 mediated regulation of hereterologous expression of Rab5a chimeric construct, pmir-GLO chimeric construct containing Rab5a 3^/^-UTR or its mutant were cotransfected with 40 nM miR-494 or control mimic miR into semiconfluent HeLa cells. After 48 h, cells were lysed and luciferase activity was measured as described in Materials and Methods. Results are represented as mean ± S.D. of three independent experiments and expressed as relative luciferase activity compared to miR-494 untreated control cells arbitrarily chosen as one unit. To determine miR-494 mediated regulation of endogenous expression of Rab5 isoforms, HeLa (B) or THP-1 differentiated human macrophages (C) were transfected with 40 nM miR-494 and levels of different isoforms of Rab5 were determined as described in Materials and Methods. Results are represented as mean ± S.D. of three independent experiments and demonstrated as relative expression of Rab5 isoforms compared to untreated control arbitrarily chosen as one unit. Inset (C) shows the level of expression of miR-494 in THP-1 transfected cells. D. THP-1 differentiated human macrophages were transfected with indicated concentrations of miR-494 and levels of Rab5a protein were determined by Western blot analysis as described in Materials and Methods. Actin was used as control. Results are represented as mean ± S.D. of three independent experiments and normalized to respective control. Expression of normalized Rab5a in uninfected cells was arbitrarily chosen as one unit. Results of control (*) and miR-494 overexpressed cells were analyzed by paired *t* test and levels of significance are indicated by *P* value.

In order to validate the regulation of Rab5a expression by miR-494, we prepared chimeric construct by ligating 3^/^-UTR of human Rab5a with luciferase as heterologous reporter. First, the human 3^/^-UTR of Rab5a was identified from human genome database and amplified (1350 bp) from cDNA prepared from THP-1 macrophage using appropriate primers. Subsequently, amplified product (1350 bp) was digested and cloned in pmir-GLO dual luciferase vector at *SacI/XhoI* restriction sites. To determine the specificity, we also made another chimeric construct of 3^/^-UTR of Rab5a containing mutation in miR-494 recognition element (5^/^-CGACACGG-3^/^). The chimeric construct containing 3^/^-UTR region of Rab5a or its mutant was co-transfected with miR-494 mimic into HeLa cells and firefly luciferase reporter activity was measured after 48 h of transfection. Co-transfection of 3^/^-UTR region of human Rab5a and a nonspecific miR mimic into HeLa cells was used as a control. The result presented in the Fig 3A showed that transfection with the miR-494 (40 nM) reduces about 50% luciferase activity of Rab5a 3^/^-UTR reporter, whereas about 20% inhibition of mutant Rab5a 3^/^-UTR reporter was detected. These results indicated that miR-494 binds with miR-494 recognition element present in the 3^/^-UTR of human Rab5a to repress the expression of Rab5a. To demonstrate that miR-494 specifically regulates the expression of Rab5a, HeLa (Fig 3B) and THP-1 (Fig 3C) cells were transfected with miR-494 mimic (40 nM) or control mimic and levels of Rab5 isoforms were determined by qPCR using specific TaqMan probes. We observed that miR-494 specifically inhibits about 50% expression of Rab5a mRNA compared to the control mimic in both cells types. No significant changes were observed in Rab5b and Rab5c levels in miR-494 transfected cells. Finally, we checked the expression of Rab5a protein after 48 h of transfection of indicated concentration of miR-494 in THP-1 macrophages by Western blot analysis using specific antibody. Our results demonstrated that overexpression of miR-494 inhibits Rab5a expression in a concentration dependent manner. More than 80% inhibition of Rab5a protein expression was observed in 100 nM miR-494 transfected human macrophages in comparison to the control miR transfected cells (Fig 3D). Similar results were also observed in HeLa cells overexpressing miR-494 (S4 Fig). These results demonstrated that miR-494 regulates the expression of Rab5a in human cell.

### *Leishmania donovani* resides in the early endosomal compartment in human macrophages by recruiting Rab5a and EEA1

**Fig 4:**
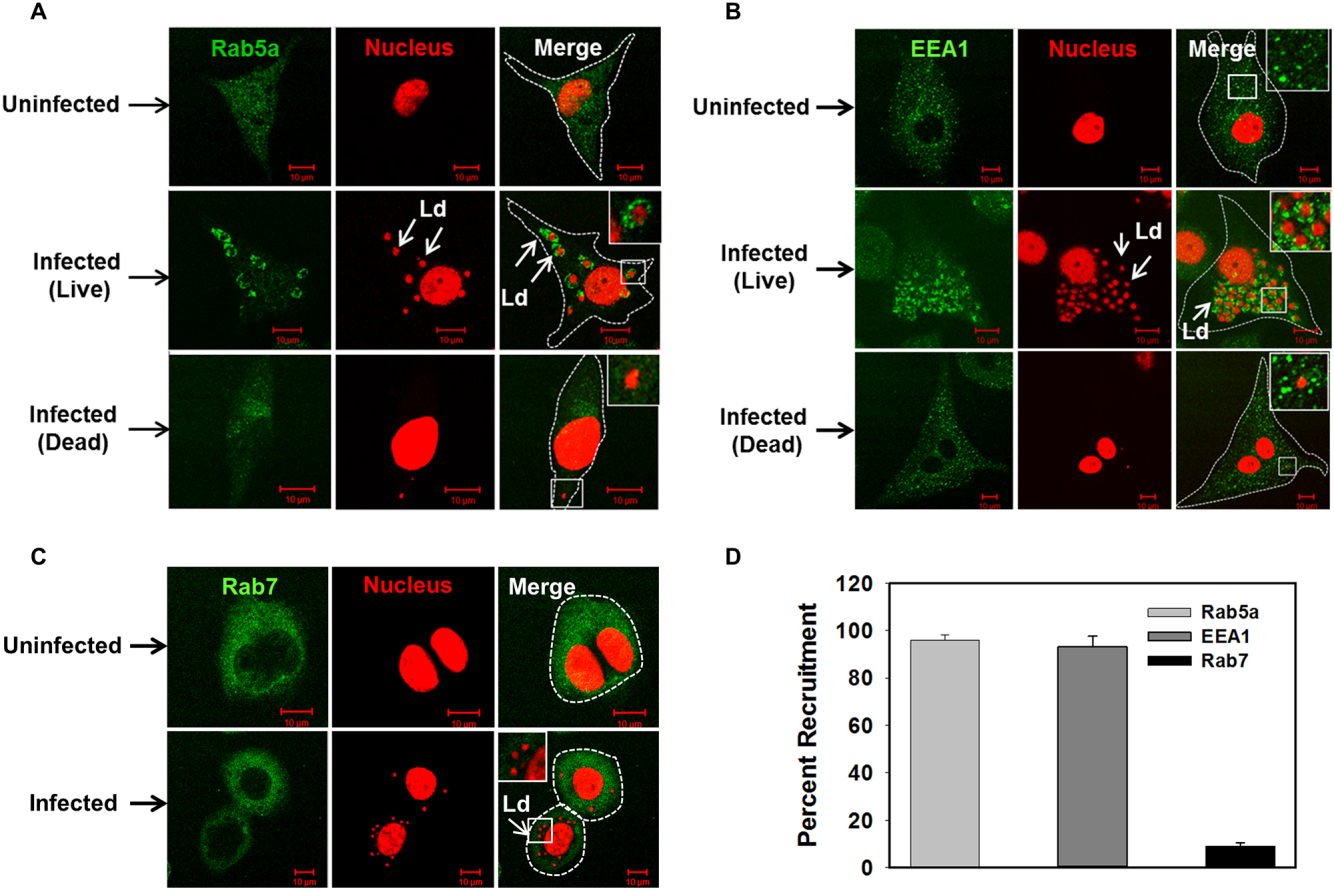
*Leishmania donovani* containing PV specifically recruits Rab5a and EEA1 in human macrophages. THP-1 differentiated human macrophages were infected with *Leishmania* and recruitment of Rab5a (A), EEA1 (B), Rab7 (C) were determined after 24 h of infection by immuno-staining with specific antibody as described in Materials and Methods. Uninfected cells were used as control. *Leishmania* and macrophage nucleus were stained with propidium iodide (Red). All results are representative of three independent experiments. D. Results are represented as mean ± S.D. of three independent experiments and expressed as percentage of *Leishmania*-PV positive for indicated markers after counting 100 cells.

**Fig 5:**
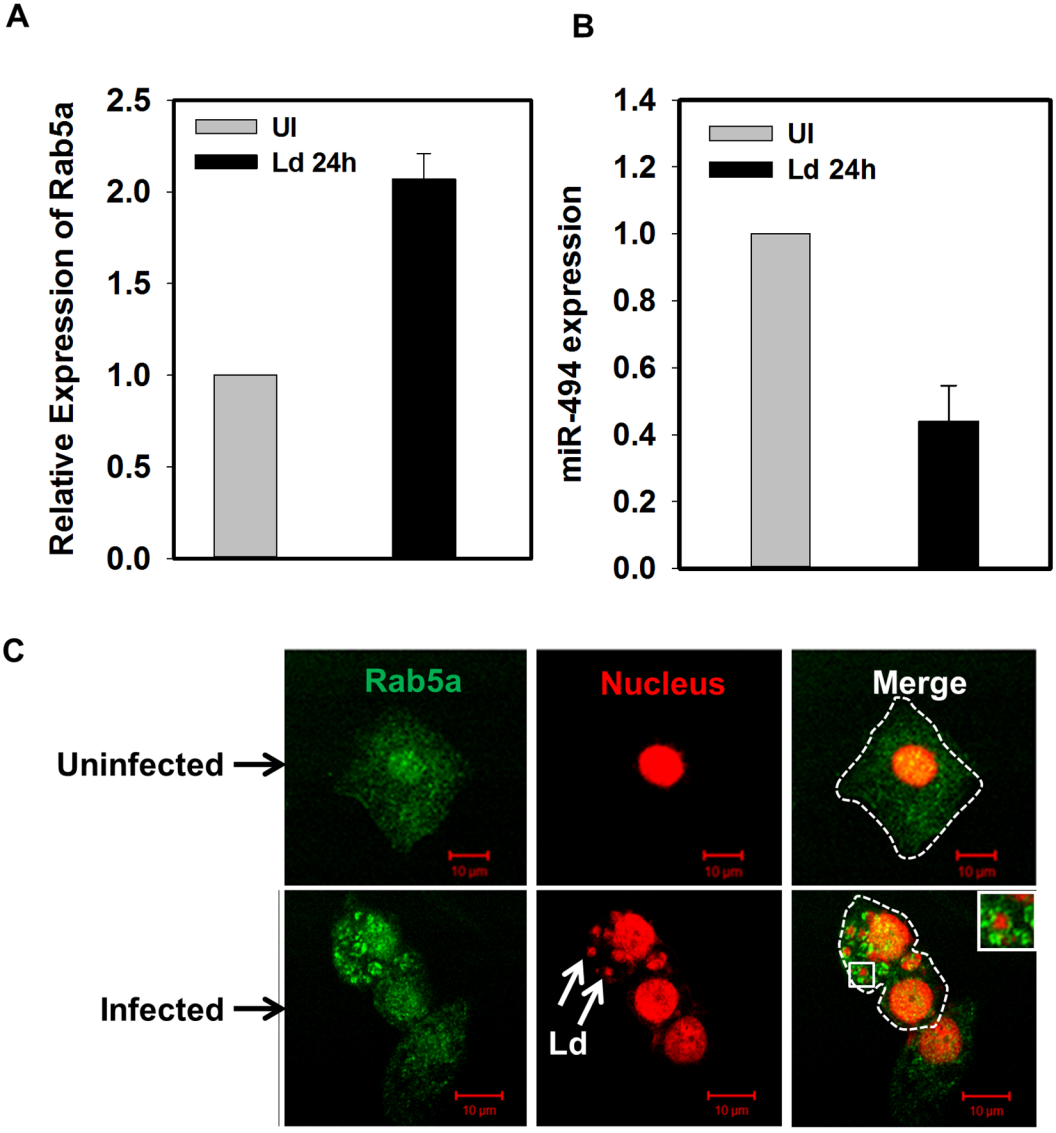
*Leishmania donovani* containing PV specifically recruits Rab5a human PBMC differentiated macrophages. A. Levels of Rab5a in infected and uninfected PBMC differentiated macrophages after 24 h of infection were determined by qPCR as described in Materials and Methods. B. Human PBMC differentiated macrophages were infected with *Leishmania* (MOI 1:20) and level of expression of miR-494 in uninfected and infected cells was determined at indicated time by qPCR as described in Materials and Methods. All results are represented as mean ± S.D. of three independent experiments and normalized to respective control. Expression of normalized respective data in uninfected cells was arbitrarily chosen as one unit. C. Human PBMC differentiated macrophages were infected with *Leishmania* and recruitment of Rab5a was determined after 24 h of infection by immuno-staining with specific antibody as described in Materials and Methods. Uninfected cells were used as control. Results are representative of three independent experiments.

Subsequently, we determined whether *L*. *donovani* recruits Rab5a on *Leishmania* containing parasitophorous vacuole (*Leishmania*-PV) in macrophages. Indeed, we found that Rab5a is specifically recruited on *Leishmania*-PV (Fig 4A) after 24 h of infection in human macrophages. In contrast, cells infected with dead parasites were unable to recruit Rab5a and it was found to be localized on discrete small punctate vesicular structures as observed in uninfected cells. Among the different isoforms of Rab5, interestingly our results showed that Rab5a is predominantly recruited (S5A Fig) and retained on *Leishmania*-PV for at least 48 h (S5B Fig). Moreover, we also found that *Leishmania*-PV also recruits (Fig 4B) and retains (S5C Fig) Early Endosome Associated Antigen (EEA1), a Rab5 effector. Moreover, we also observed higher recruitment of Rab5a and EEA1 on PV at 24 h in comparison to 6 h. In addition, results presented in the Fig 4C showed that *Leishmania*-PV does not recruit Rab7 possibly to block transport to lysosomes. We also used anti-Rab8 and anti-Rab9 antibodies as control and found that *Leishmania*-PV does not recruit Rab9 and Rab8 indicating that Rab5a recruitment on PV is specific (S5D Fig). Further quantitation revealed that more than 90% of *Leishmania*-PV recruits Rab5a and EEA1 whereas less than 10% PV recruits Rab7 (Fig 4D). In addition, our results also showed that *L*. *donovani* infection overexpresses Rab5a in human PBMCs (Fig 5A) by downregulating the expression of miR-494 (Fig 5B). Consequently, Rab5a was found to be recruited on *Leishmania*-PV in *Leishmania* infected human PBMCs (Fig 5C). In contrast, we observed that *Leishmania* infection in Raw 264.7 mouse macrophages neither induces the expression of Rab5a (S6A Fig) nor recruits Rab5a on *Leishmania*-PV (S6B Fig). Taken together, these results demonstrated that *Leishmania*-PV specifically recruits and retains Rab5a and EEA1 in human macrophages to reside in an early endocytic compartment.

### *Leishmania* inhibits its transport to lysosomes in macrophages

**Fig 6:**
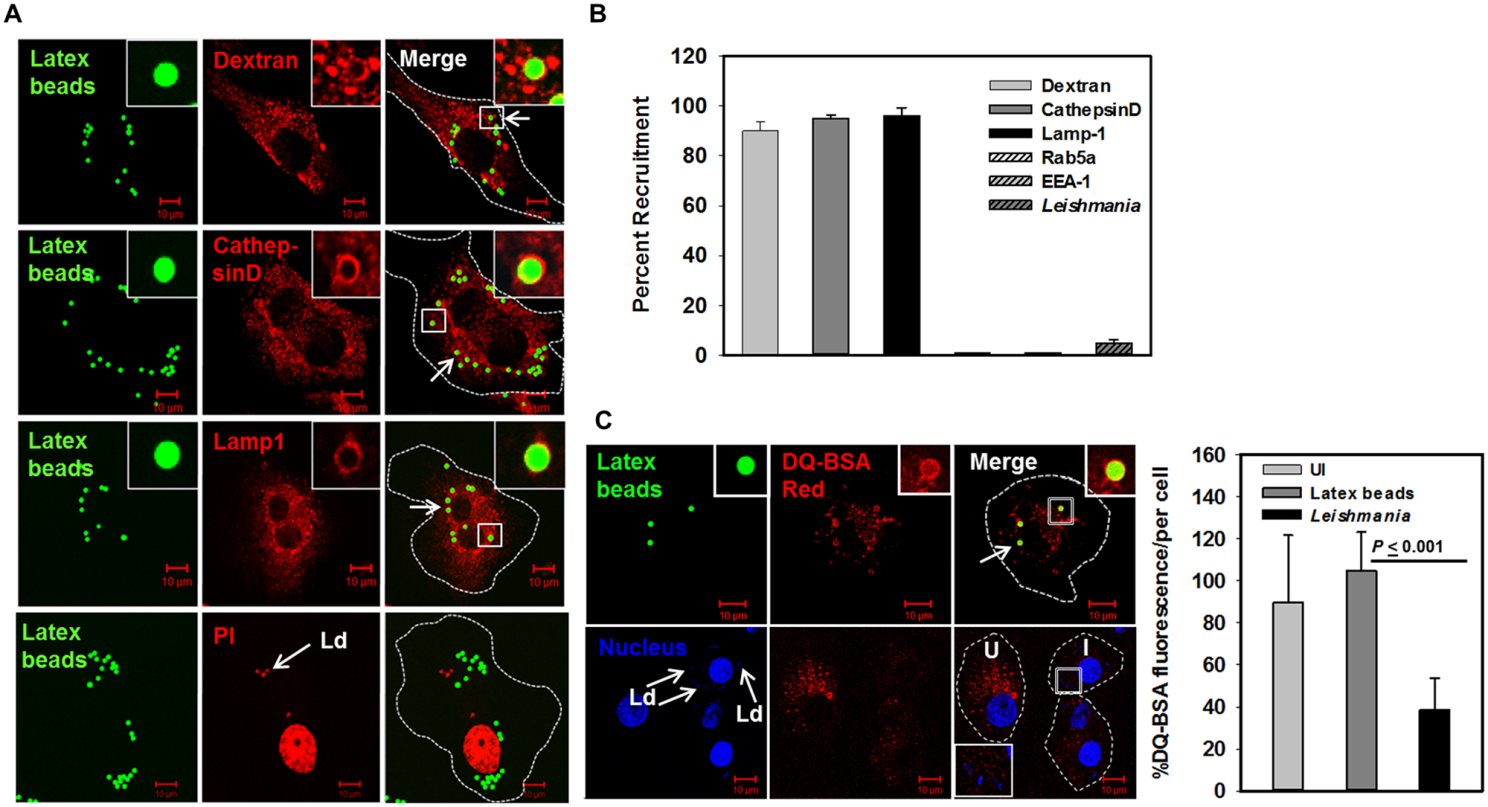
*Leishmania donovani* inhibits its transport to lysosomes in macrophages. A. Lysosomes of THP-1 differentiated human macrophages were prelabeled with internalization of Dextran Texas Red for 24 h and subsequently, cells were infected with Fluoresbrite-YG-latex beads (Green) as described in Materials and Methods (upper panel). Similarly, lysosomes of THP-1 differentiated human macrophages were labeled with Green fluorescent labeled latex beads and cells were immune-stained with anti-CathepsinD (middle panel) or anti-Lamp1 (middle panel) antibody as described in Materials and Methods. THP-1 differentiated human macrophages were coinfected with *Leishmania* and Green fluorescent labeled latex beads and chased for 24 h at 37°C and their localization was determined by confocal microscopy (lower panel). *Leishmania* and macrophage nucleus were stained with propidium iodide (Red). All results are representative of three independent experiments. B. Results are represented as mean ± S.D. of three independent experiments and expressed as percentage of latex-bead containing phagosomes positive for indicated markers after counting 100 cells. C. THP-1 differentiated human macrophages were infected with *Leishmania* or Green fluorescent labeled latex beads. Cells were incubated for 24 h at 37°C and lysosomes were labeled with DQ-BSA Red as described in Materials and Methods. *Leishmania* and macrophage nucleus were stained with Hoechst (Blue). Cells were viewed in an LSM 510 Meta confocal microscope using an oil immersion objective. Results are expressed as mean percentage of total fluorescence per cell ± S.D. of 100 independent indicated cells.

In order to unequivocally prove that *Leishmania* inhibits its transport to lysosomes by recruiting Rab5a and EEA1 on PV, lysosomes of the human macrophages were labeled with the internalization of latex beads and infected with *Leishmania*. Accordingly, THP-1 differentiated human macrophages were incubated with Fluoresbrite-YG-latex beads (2 μm) for 3 h and chased for 24 h at 37°C and subsequently cells were immuno-stained with anti-Lamp1 or anti-CathepsinD antibody. As expected, our results showed that latex bead containing phagosomes after 24 h of internalization are positive for both Lamp1 and CathepsinD (Fig 6A). In addition, latex beads after 24 h of internalization in human macrophages were found to be transported to dextran-Texas Red prelabeled lysosomes (Fig 6A). These results clearly demonstrated that lysosomes in macrophages can be labeled with internalization of latex beads for 24 h. Therefore, THP-1 differentiated macrophages were co-infected with latex beads and live *Leishmania* for 3 h at 37°C, washed and chased for additional 24 h at 37°C. Our results showed that *Leishmania*-PVs are clearly separated from latex beads containing lysosomes in human macrophages after 24 h of incubation (Fig 6A, lower panel). In addition, we also found that latex beads containing phagosomes are clearly separated from Rab5a and EEA1 positive *Leishmania*-PV in macrophages after 24 h of infection (S7 Fig). Further quantitation revealed that more than 90% of the latex beads containing phagosomes are positive for Dextran-Texas Red, Lamp1 and CathepsinD whereas Rab5a and EEA1 were almost not detected on these phagosomes after 24 h of internalization in macrophages. Most interestingly, more than 95% of *Leishmania*-PVs were found to be separated from Latex beads containing lysosomes under same conditions (Fig 6B).

To determine the proteolytic activity of the lysosomes of the *L*. *donovani* infected and uninfected human macrophages, cells were labeled with DQ-BSA Red which induces strong fluorescence upon hydrolysis by proteases [29]. Our results showed that most of the internalized latex beads in human macrophages are colocalized with DQ-BSA Red labeled proteolytically active lysosomes. Whereas, *Leishmania* failed to colocalize with DQ-BSA Red labeled proteolytically active lysosomes (Fig 6C). Most importantly, a significant reduction in the fluorescence of DQ-BSA Red and numbers of red puncta per cell were observed in *Leishmania* infected human macrophages in comparison to uninfected and latex beads infected cells (Fig 6C). These results indicated that *Leishmania* not only inhibits transport to lysosomes but also blocks the proteolytic activity of the lysosomes in infected macrophages.

### *Leishmania donovani* recruits immature forms of lysosomal proteins on their parasitophorous vacuoles

**Fig 7:**
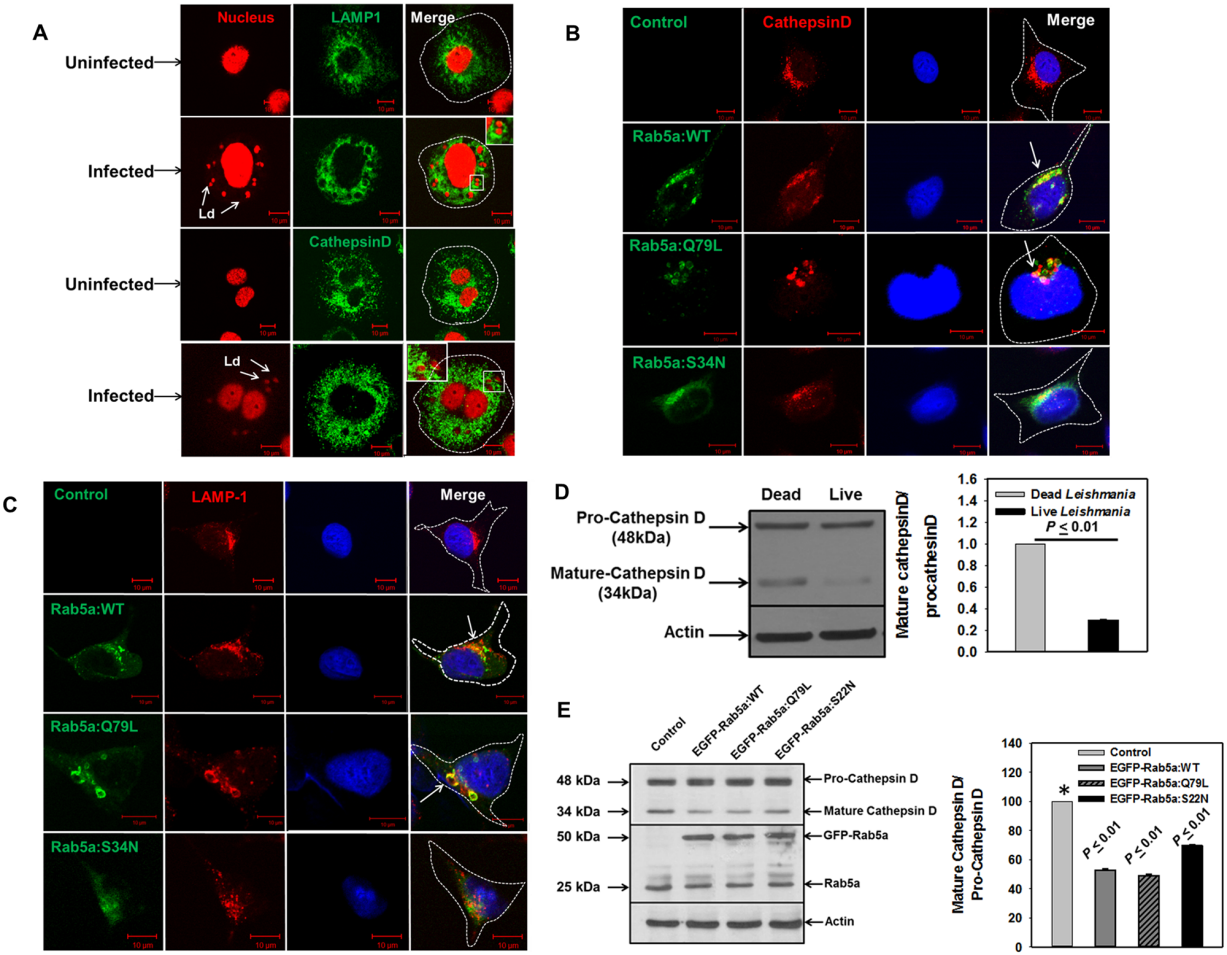
Recruitment of Lamp1 and CathepsinD on *Leishmania donovani* containing PV. A. THP-1 differentiated human macrophages were infected with *Leishmania* and recruitment of Lamp1 and CathepsinD were determined after 24 h of infection by immuno-staining with specific antibody as described in Materials and Methods. Uninfected cells were used as control. *Leishmania* and macrophage nucleus were stained with propidium iodide (Red). B. Rab5a or its mutants were overexpresses in HeLa cells as GFP fusion protein and cells were immuno-stained with anti-CathepsinD or anti-Lamp1 antibody (C). Cells were viewed under confocal microscope to determine the localization of indicated protein. All results are representative of three independent experiments. D. THP-1 differentiated human macrophages were infected with live or dead *Leishmania* and chased for 24 h. Finally, cells were lysed and size of the CathepsinD was determined by Western blot analysis using specific antibody. Results are represented as mean ± S.D. of three independent experiments and expressed a ratio of mature CathepsinD and pro-CathepsinD compared to ratio in dead infected cells arbitrarily chosen as one unit. Results of dead and live parasite infected cells were analyzed by paired *t* test and levels of significance are indicated by *P* value. E. Similarly, size of the CathepsinD was determined in Rab5a:WT, Rab5a:Q79L or Rab5a:S34N overexpressed HeLa cells using specific antibody. Results are represented as mean ± S.D. of three independent experiments and expressed as a ratio of mature CathepsinD and pro-CathepsinD compared to ratio in control cells normalized to actin was arbitrarily chosen as one unit.

Previous studies demonstrated that *Leishmania* resides in the phagolysosomal compartment in mouse macrophages [30,31]. However, we found that *Leishmania* resides in Rab5a and EEA1 positive early endocytic compartment in human macrophages. Therefore, we tried to determine the localization of CathepsinD and Lamp1 on *Leishmania*-PV after 24 h of infection in human macrophages. Interestingly, we also found that *Leishmania*-PV acquires Lamp1 and CathepsinD after 24 h of infection in human macrophages (Fig 7A) as observed previously in mouse macrophages. This observation was puzzling; therefore, we tried to determine how *Leishmania*-PV recruits lysosomal markers like CathepsinD and Lamp1 when parasites reside in an early compartment positive for Rab5a and EEA1. Previous studies demonstrated that lysosomal enzymes like CathepsinD and Lamp1 are trafficked via early endosomes to their final destination of lysosomes [32]. Thus, we speculated that induced expression of Rab5a in *Leishmania* infected macrophages might promote the fusion of Golgi derived Lamp1 or CathepsinD containing vesicles with *Leishmania* PV and thereby, blocks the transport of lysosomal proteins in early endosomes. To test this hypothesis, we overexpressed GFP-Rab5a or its mutants in HeLa cells and determined the distribution of CathepsinD and Lamp1 by immune-staining. Our results showed that both CathepsinD (Fig 7B) and Lamp1 (Fig 7C) are localized into perinuclear lysosomal compartment in untransfected control cells as reported earlier [33]. In contrast, majority of CathepsinD (Fig 7B) and Lamp1 (Fig 7C) containing vesicles were found to be colocalized with GFP-Rab5a positive early endosomal compartments in GFP-Rab5a:WT and GFP-Rab5a:Q79L overexpressed cells. No apparent change in the distribution CathepsinD and Lamp1 was observed in GFP-Rab5a:S34N overexpressing HeLa cells in comparison to control cells. These results demonstrated that overexpression of Rab5a inhibits the transport of CathepsinD and Lamp1 to the lysosomes and retains them in the early endocytic compartment.

Previous studies demonstrated that the lysosomal hydrolase CathepsinD is synthesized as a 52-kDa precursor protein which is cleaved from amino-terminus in acidified early endosome resulting in a 48-kDa intermediate enzyme form. Subsequently, further proteolytic cleavage of the protein in low pH of the lysosomes produced the mature active CathepsinD composed of heavy (34-kDa) and light (14-kDa) chains linked by non-covalent interactions [34]. Thus, size of CathepsinD was used as an indicator for their processing as well as for their localization in previous studies. Therefore, we determined the size of the CathepsinD in *Leishmania* infected human macrophages. We found significantly lower levels of the mature/activated form of CathepsinD in live parasite infected macrophages in comparison to dead parasite infected cells (Fig 7D). Further quantitation revealed that live *Leishmania* infection inhibits about 70% mature form of CathepsinD in infected human macrophages than dead parasite infected cells. Similar block in the processing of CathepsinD was found in Rab5a:WT, Rab5a:Q79L and Rab5a:S34N overexpressing HeLa cells (Fig 7E). Taken together, these results clearly demonstrated that *Leishmania* induces the expression of Rab5a in infected human macrophages and thereby blocks the trafficking of lysosomal proteins and retains them as inactive precursor form in early endosomes.

### Rab5a function is necessary for the survival of *Leishmania donovani* in human macrophages

**Fig 8:**
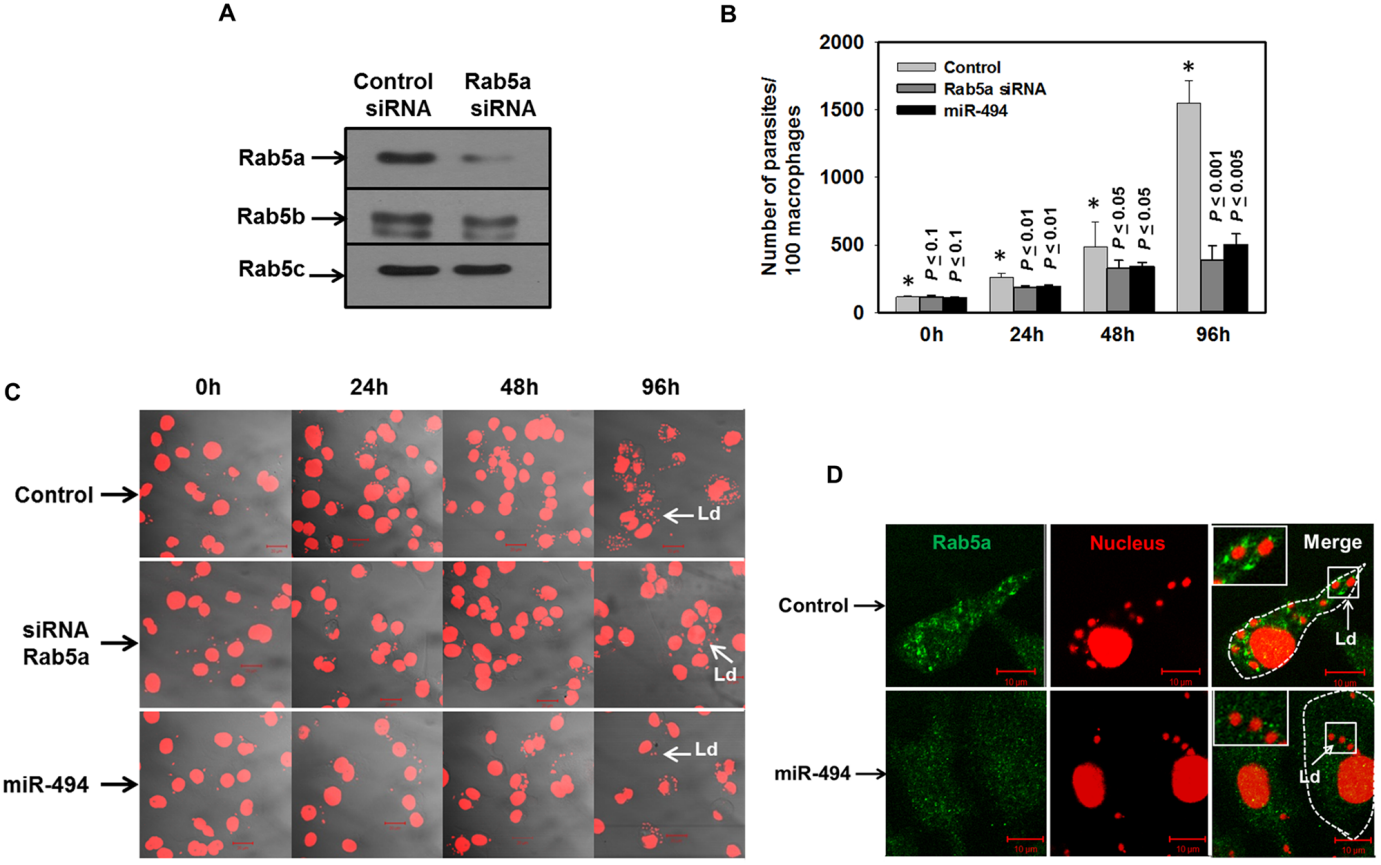
Rab5a function is required for the survival of parasites in macrophages. A. THP-1 differentiated human macrophages were transfected with 50 nM Rab5a specific siRNA or control RNA and levels of different isoforms of Rab5 were determined after 48 h by Western blots analysis. B. THP-1 differentiated human macrophages were transfected with 50 nM miR-494 or Rab5a specific siRNA and subsequently cells were infected with *Leishmania* promastigotes as described in Materials and Methods. Parasites load in the infected macrophages were microscopically estimated at indicated time. Results are expressed as numbers of parasites present in 100 macrophages ± S.D. from three independent experiments. Results of control (*) and Rab5a siRNA/miR-494 overexpressed cells were analyzed by paired *t* test and levels of significance are indicated by *P* value. C. Confocal images of the representative experiment showing the parasite load in macrophages. *Leishmania* and macrophage nucleus were stained with propidium iodide (Red). D. THP-1 differentiated human macrophages were transfected with 50 nM miR-494 and infected with *Leishmania* for 24 h as described in Materials and Methods. Recruitment of Rab5a on *Leishmania*-PV in miR-494 overexpressed human macrophages was detected using specific antibody by confocal microscopy. Results are represented of three independent experiments.

To determine the function of Rab5a in the survival of *L*. *donovani* in human macrophages, Rab5a was knockdown in THP-1 differentiated macrophages by specific siRNA and these cells were infected with *Leishmania*. First, THP-1 differentiated macrophages were transfected with 50 nM of Rab5a specific siRNA and cells were incubated for 48 h at 37°C. Subsequently, levels of Rab5 isoforms in these cells were determined by Western blot analysis. The results presented in the Fig 8A showed that siRNA specifically downregulates the expression of Rab5a in macrophages. Finally, Rab5a knockdown cells were infected with *Leishmania* and the parasite load was determined at indicated time points. Our results showed that infection in Rab5a knockdown macrophages is not compromised as similar numbers of parasites are observed in both Rab5a knockdown and control cells at 0 h. However, more than 74% inhibition of parasite load was observed in Rab5a knockdown human macrophages in comparison to control cells after 96 h of infection (Fig 8B and 8C). Similar results were also obtained in miR-494 (50 nM) overexpressed macrophages under identical conditions (Fig 8B and 8C). Subsequently, our results showed that this is due to significant less recruitment of Rab5a on *Leishmania*-PV in miR-494 overexpressed human macrophages (Fig 8D). These results demonstrated that knocking down of Rab5a by siRNA or inhibiting the expression of Rab5a by miR-494 significantly inhibits the growth of parasite in macrophages.

## Discussion

One general strategy used by several intracellular pathogens is to avoid transport to lysosomes by modulating the intracellular trafficking pathway in the host cells to survive in modified phagosomes [11,12,13]. In contrast, previous studies using mainly mouse macrophages have shown that *Coxiella* [35,36] and *Leishmania* [2,3] survive in a phagolysosomal compartment decorated with Lamp1, vacuolar ATPase, and CathepsinD. As phagolysosomal compartment is detrimental for invading pathogen, it is puzzling how *Coxiella* and *Leishmania* are surviving in such a degradative compartment in the cell. Consequently, recent studies have shown that pathogenic *C*. *burnetii* blocks the recruitment of Vps41 and inhibits its transport to phagolysosomes [37,38]. However, not much is known about how *Leishmania* modulates the trafficking pathway in the host cells and the nature of the compartment they survive.

Interestingly, we have found that *Leishmania donovani* infection specifically upregulates the expression of Rab5a in human macrophages. Subsequently, we have shown that upregulation of Rab5a expression is due to transcriptional activation as enhanced level of Rab5a transcript is detected in infected macrophages. To understand how *Leishmania* induces the expression of Rab5a in infected human macrophages, we have compared the miRNA profile of uninfected and infected macrophages as some of the current studies have shown that miRNA can also modulate the expression of Rab GTPases [39,40]. In addition, it has been shown that *Leishmania* infection downregulates miR-122 expression to lower serum cholesterol [41] and significantly enhances the miR-30A-3p expression to modulate autophagic pathway in macrophages [42]. Interestingly, we have also found that *L*. *donovani* infection modulates the expression of various miRNA in human macrophages. Subsequently, we have identified that miR-494 has a target site in 3^/^-regulatory region of Rab5a of human and hamster but not in mouse. As not much work has been done about regulation of expression of Rab GTPases, we have first validated that miR-494 specifically regulates the expression of Rab5a using a chimeric construct containing 3^/^-UTR of Rab5a with luciferase as heterologous reporter in mammalian cells. Our results have demonstrated that miR-494 binds with miR-494 recognition element present in the 3^/^-UTR of Rab5a to repress the expression of Rab5a in human cells. Most importantly, we have found that transfection of miR-494 specifically inhibits the expression of Rab5a protein in human macrophages and HeLa cells compared to the control mimic. This is the first demonstration that miR-494 negatively regulates the expression of Rab5a in HeLa cells and macrophages.

Subsequently, we have shown that *L*. *donovani* infection downregulates the expression of miR-494. To determine how *L*. *donovani* infection downregulates the expression of miR-494 in macrophages, we have checked the expression of AP-1 transcription factor in infected human macrophages as it has been previously reported that AP-1 transcription factor is involved in the synthesis of miR-494 [25]. Interestingly, we have found that *Leishmania* degrades the c-Jun subunit of AP-1 transcription factor by gp63 in infected human macrophages using gp63 secretion deficient LdRab1:S22N transgenic parasites [28]. These results are supported by the fact that *Leishmania major* infection degrades AP-1 complex in infected mouse macrophages via their metalloprotease gp63 [26]. Consequently, we have also found that gp63 overexpression degrades c-Jun in Raw 264.7 mouse macrophages. Taken together, our results have demonstrated that *L*. *donovani* infection degrades c-Jun by their metalloprotease, gp63 to inhibit the synthesis of miR-494 and thereby upregulates the expression of Rab5a in infected human macrophages. In contrast, *Leishmania* infection does not induce the expression of Rab5a in mouse macrophages as miR-494 target site is absent in the 3^/^-UTR of mouse Rab5a.

In order to understand the significance of Rab5a upregulation in *L*. *donovani* infected human macrophages, we have checked the recruitment of Rab5a on *Leishmania*-PV. We have found that *Leishmania* not only specifically recruits Rab5a on PV but also retains it throughout the experimental period of 48 h. Similarly, *Leishmania* recruits and retains EEA1 on its PV which is a Rab5 effector molecule present in early endosomal compartment [43]. However, *Leishmania* containing PV does not acquire Rab7, Rab8 and Rab9 in human macrophages. It is also pertinent to mention that antibodies against several mammalian Rabs like Rab5, Rab7 and Rab1 do not cross react even with respective *Leishmania* proteins as it has been shown previously [28,44,45,46]. Therefore, our results unequivocally prove that *Leishmania* resides in the early endocytic compartment in human macrophages by selectively recruiting Rab5 and EEA1 as observed for *Mycobacteriun* and *Salmonella* -containing phagosomes [15,47,48]. As Rab5 is an early endocytic GTPase [49,50], retention of Rab5a on PV might promote the constitutive fusion of *Leishmania*-PV with early endosomes to inhibit its trafficking to lysosomes as it has been shown previously with *Salmonella*-containing phagosomes [51]. Therefore, we have checked whether *Leishmania* inhibits its transport to lysosomes. Indeed, we have found that *Leishmania* inhibits its transport to latex beads containing lysosomes even after 24 h of infection in human macrophages. Consequently, we have found that *Leishmania*-PV does not recruit Rab7 which is required for lysosomal targeting. Taken together, these results unambiguously prove that *Leishmania* inhibits its transport to the lysosomes to survive in human macrophages. However, our results have also shown that *Leishmania* containing PV in mouse macrophages does not recruit and retain Rab5a after 24 h of infection whereas *Leishmania* containing PV in mouse macrophages is shown to recruit Rab5 in early time point of infection [52]. This is possibly due to the fact that *Leishmania* infection does not overexpress Rab5a in mouse macrophages. Conversely, our results also suggest that *Leishmania* infection in hamster macrophages might induce the Rab5a expression to reside in early endosomes to produce persistent infection. Thus, it is tempting to speculate why among the two animal models of leishmaniasis [53], hamster model mimic human infection whereas *Leishmania* infection is self-healing in mouse.

However, it has been previously shown that *Leishmania*-PV recruits lysosomal markers like CathepsinD and Lamp1 predominantly in mouse macrophages [30,31]. We have confirmed these observations and have also found that *Leishmania*-PVs are positive for CathepsinD and Lamp1 in human macrophages. This is puzzling how *Leishmania*-PV recruits lysosomal markers like CathepsinD and Lamp1 when they reside in an early compartment positive for Rab5a and EEA1. Thus, we have evaluated the trafficking of CathepsinD and Lamp1 in Rab5a overexpressed cells as *Leishmania* infection induces the expression of Rab5a in the infected cells. Our results have shown that both Lamp1 and CathepsinD are predominantly colocalized with GFP-Rab5a positive early endosomal compartments in GFP-Rab5a:WT and GFP-Rab5a:Q79L overexpressed cells in comparison to their perinuclear localization in control cells as reported earlier [33]. Thus, overexpression of Rab5a might promote the fusion of Golgi derived Lamp1 or CathepsinD containing vesicles with early endosomes and thereby, retain them in early endosomes as these proteins are trafficked via early endosomes to lysosomes [54]. Our results are also supported by the fact that overexpression of dominant active mutant of Rab5a redistributes the lysosomal enzymes in early endosomes and disturbs the lysosome biogenesis [55]. These results suggest that higher expression of Rab5a in *L*. *donovani* infected human macrophages blocks the trafficking of lysosomal enzymes and retains them in early endocytic compartment. Moreover, it has been shown that size of CathepsinD present in early endosomal compartment is 48kDa whereas the size of the mature protein in the lysosome is found to be around 34 kDa and 14 kDa [56]. Consequently, we have found that size of the CathepsinD is predominantly 48 kDa in *L*. *donovani* infected human macrophages as well as Rab5a and its mutants overexpressed cells indicating that *Leishmania* infection retains lysosomal enzymes in early compartment in an immature and inactive form via the overexpression of Rab5a. Though overexpression of Rab5a:S34N does not apparently alter the distribution of CathepsinD in HeLa cells, but our results have shown that overexpression of this mutant also blocks the processing of this enzyme. This might be due to the essential role of Rab5 in the biogenesis of the endolysosomal system [57]. In addition, we have shown that *L*. *donovani* infection blocks proteolytic activity of the lysosomes in infected human macrophages using DQ-BSA Red as a fluorogenic substrate for proteases [29]. Thus, *Leishmania*-PV recruits Lamp1 and CathepsinD as it has been shown earlier but these proteins are localized in immature and inactive form in early endosomes.

Finally, we have shown that selective depletion of Rab5a in human macrophages by specific siRNA significantly inhibits the growth of the parasites. Similar results are also obtained in miR-494 overexpressed macrophages under identical conditions. These results suggest that knocking down of Rab5a by siRNA or inhibiting the expression of Rab5a by miR-494 possibly targets internalized parasites to lysosomes as they will not be able to promote Rab5a-mediated constitutive fusion with early endosomes. Thus, our results demonstrate that Rab5a function is essential for the survival of *Leishmania* in human macrophages.

**Fig 9:**
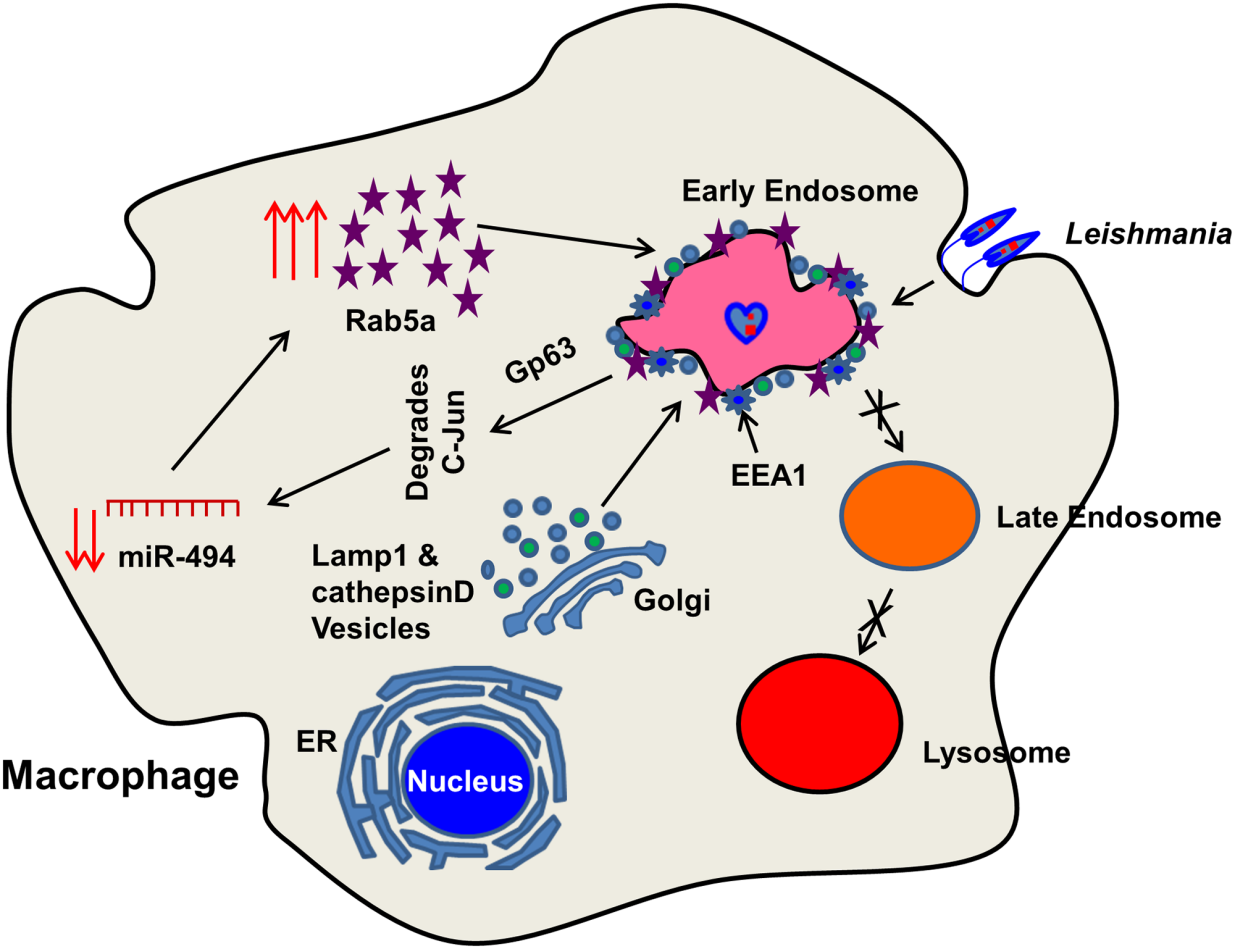
Schematic representation of mechanism of survival of *Leishmania donovani* in early endocytic compartment in macrophages. *Leishmania* after entering into human macrophages secretes gp63 which degrades c-Jun to downregulate the expression of miR-494 and thereby overexpress Rab5a in the infected cells. Subsequently, *Leishmania* recruits and retains Rab5a, EEA1 on PV and inhibits transport to lysosome. Interestingly, *Leishmania* also recruits inactive CathepsinD and Lamp1 on PV to reside in a modified early endocytic compartment in human macrophages.

In conclusion, this is the first demonstration that *Leishmania* resides in Rab5a and EEA1 positive early endocytic compartment in human macrophages. To delineate the mechanism, we have shown that *Leishmania* upregulates the expression of Rab5a in infected macrophages by downregulating the synthesis of miR-494 by degrading c-Jun via gp63. Subsequently, parasites recruit Rab5a on PV and inhibit transport to lysosomes (Fig 9). However, *Leishmania* residing in early compartment also recruits lysosomal enzymes in immature and inactive form in human macrophages. Thus, blocking the processing of the lysosomal enzymes to the mature active form might also help the parasites to survive in macrophages. These results also indicate the possibility of modulating endo-lysosomal pathway in parasite infected cells by miR-494 or small molecules to divert trafficking of *Leishmania* probably to lysosome which might be useful for developing future therapeutic intervention.

## Materials and methods

### Materials

Unless otherwise stated, all reagents were obtained from Sigma Chemical Co. (St. Louis, MO). Tissue culture supplies were obtained from the Grand Island Biological Co. (Grand Island, NY). Lipofectamine 2000 and Lipofectamine RNAi max reagent were purchased from Thermo Fisher Scientific. pmirGLO Dual-Luciferase miRNA Target Expression Vector and Dual-Luciferase Reporter Assay System were purchased from Promega Life Science (Madison, WI). microRNA mimics were obtained from Sigma Aldrich (St. Louis, MO). Antibodies against Rab5a and Rab5c were purchased from Abcam (Cambridge, England) whereas anti-Rab5b antibody was obtained from Santa Cruz Biotechnology (Santa Cruz, CA). Antibodies against Rab5, Rab7, Rab9, c-Jun, Lamp1 were obtained from Cell Signaling Technologies (Danver, MA). Anti-Rab8 and anti-Rab11 antibodies were purchased from BD Biosciences, USA. Anti-EEA1 antibody was received as kind gift from Dr. Marino Zerial (Max Planck Institute, Dresden, Germany). All HRP-conjugated secondary antibodies were purchased from Jackson ImmunoResearch Laboratory (West Grove, PA) and ECL was obtained from Amersham Biosciences, UK. All secondary antibodies used for immunofluorescence studies were purchased from Molecular Probes (Eugene, OR). All other reagents used were of analytical grade.

#### Cells

Human acute monocytic leukemia-derived cell line (THP-1; Cat. No. TIB-202) and Raw 264.7 mouse macrophages cell line (Cat. No.TIB-71) were obtained from American Type Culture Collection, Manassas, Virginia. Cells were routinely cultured in complete RPMI (RPMI-1640 containing 10% FCS and 50 μg/ml gentamycin) at 37°C in a humidified incubator with 5% CO_2_. THP-1 cells were differentiated into macrophages in the presence of Phorbol 12-myristate 13-acetate (100ng/ml) for 24h. Cells were washed and incubated for another 24 h in complete RPMI without PMA and used for experimental purpose. Human epithelial carcinoma cell line (HeLa) was obtained from American Type Culture Collection (Cat. No. CCL-2), Manassas, Virginia. The cells were maintained in complete DMEM medium containing 10% FCS, pH 7.4 at 37°C in a humidified incubator with 5% CO_2_. Peripheral blood mononuclear cells (PBMCs) were isolated from heparinized venous blood from healthy human volunteers using a Ficoll Paque density gradient. For differentiation into macrophages, isolated PBMCs were incubated with M-CSF (5 ng/ml) for 7 days at 37°C in a humidified incubator with 5% CO_2_.

#### Infection

*Leishmania donovani* Bob strain (LdBob strain/MHOM/SD/62/1SCL2D) was used for infection. Promastigotes were routinely grown in M199 medium (Gibco) buffered with HEPES (40mM) containing 10% FCS, 50 μg/ml gentamicin and Hemin (10 μg/ml) at 23°C. Parasites were repeatedly passaged in BALB/c mice to maintain the virulence. Freshly transformed promastigotes from isolated amastigotes were used for infection.

THP-1 differentiated human macrophages and human PBMC differentiated macrophages were infected with *Leishmania* promastigotes in RPMI medium at MOI of 1:20 for 3 h at 37°C followed by 3 washes with plain RPMI to remove uninternalized parasites. Infected cells were incubated in complete RPMI at 37°C for indicated time points.

#### Detection of levels of various proteins in infected and uninfected cells

To detect the levels of various proteins in *Leishmania* infected and uninfected cells, cells were lysed using cell lysis buffer (1% Triton X-100 in PBS containing protease inhibitors cocktail) for 60 min at 24°C and centrifuged at 15000 x g for 15 min. Subsequently, cellular proteins (40 μg) were resolved on a SDS–PAGE and Western blot analyses were carried out using specific antibodies. All blots were quantified using ImageJ software.

#### Detection of expression of different Rabs in infected and uninfected macrophages by Real-time PCR

To compare the levels of different Rabs in infected and uninfected human macrophages, cells were lysed using TRIzol reagent and total RNA was isolated using standard procedure. Subsequently, cDNA was synthesized using Thermo Script RT-PCR kit (Invitrogen) according to the manufacturer’s instructions. Real-time PCR (qPCR) was carried out using respective Rab specific forward and reverse primers and 18s rRNA as an internal control. The final volume of reaction mixture for qPCR was 35μl containing 17.5μl of SYBR Green, 3 μl of cDNA and 1 μl of each forward and reverse primer. The samples were run in triplicate in an ABI 7500 Fast Real-Time PCR detection system using following thermal conditions: 50°C for 20 sec followed by 10 min at 95°C, then 40 cycles at 95°C for 15 sec and 60°C for 1 min. The results were analyzed using comparative Ct method (2^-ΔΔCt^). The respective gene amplification was normalized using 18s rRNA as an internal control and expressed as relative unit compared to uninfected control arbitrarily chosen as 1 unit. Primer sequences used in qPCR are indicated below

1. hRab5a: Forward 5^/^-TATTGGCCCCTTGAATTCTG-3^/^ and Reverse 5^/^-TTAGAAAAGCAGCCCCAATG-3^/^;
2. hRab5b: Forward 5^/^-TTTTCTCCTCTCCCCAGGAT-3^/^ and Reverse 5^/^-AGATCTTGCCTCCCCATTCT-3^/^;
3. hRab5c: Forward 5^/^-GTGAATGACCCGACTGGAAT-3^/^ and Reverse 5^/^-AGGGAAAATGGGAGAGCAGT-3^/^;
4. hRab7: Forward 5^/^-GCGGAGCTTTTTCCTCTTTT-3^/^ and Reverse 5^/^-TTTTATTGGCATTGCGTTCA-3^/^;
5. hRab4: Forward 5^/^-AGGACCTGGATGCAGATCGT-3^/^ and Reverse 5^/^-TCCCCTGTGAGCGCACTT-3^/^;
6. hRab11: Forward 5^/^-CGTGGAGCTGTAGGTGCCTTA-3^/^ and Reverse 5^/^-TATCAGCATGATCTCTCAGTTCTTTCA-3^/^;
7. h18s rRNA: Forward 5^/^-CGAAAGCATTTGCCAAGAAT-3^/^ and Reverse 5^/^-AGTCGGCATCGTTTATGGTC-3^/^

#### Whole-genome microarray analysis

Microarray analysis was done using total RNA isolated from *Leishmania* infected and uninfected THP-1 differentiated human macrophages from commercial facility (Genotypic Technology, Bengaluru, India). The RNA was labeled with pCp-Cy3 dye using Agilent miRNA labeling and hybridization kit (Cat # 5190–0408). The hybridization experiments were carried out at 55°C for 20 h using human miRNA 8x15k arrays (Agilent Technologies, AMADID: 029297). The microarray scanning was performed on a G2505C scanner (Agilent Technologies, G2565CA) and image was analyzed using Agilent Feature Extraction software Version 10.7 (Agilent Technologies). Differential expression was analyzed using Agilent GeneSpring GX version 11.5 software (Agilent Technologies). Normalization of the data was done in GeneSpring GX using the 90^th^ percentile shift and normalization to specific samples. Relative expression of miRNAs in infected cells was determined in comparison to uninfected control.

#### Quantitation of miR-494 in infected and uninfected macrophages

To determine the levels of expression of miR-494 in infected and uninfected cells, qPCR was done using TaqMan miRNA assays (Life Technologies, 4427975). Briefly, miRNAs were reverse transcribed to cDNA using TaqMan miRNA Reverse Transcription kit (Life Technologies, 4366596). The qPCR was carried out using master mix containing 10 μl of TaqMan Universal PCR master mix, 1 μl of the miRNA specific primers, 1.33 μl of cDNA, and final volume was adjusted to 20 μl with nuclease-free water. U6 snoRNA amplification was used as the internal control (U6 small nucleolar RNA, Life Technologies, 4427975). The PCR was carried out in triplicate in an ABI 7500 Fast Real-Time PCR detection system using following thermal conditions: 10 min at 95°C followed by 40 cycles at 95°C for 15 sec and extension for 1 min at 60°C. Results were analyzed using comparative Ct method (2^-ΔΔCt^) and expressed as relative expression compared to uninfected control arbitrarily chosen as 1 unit.

#### Luciferase assay to determine the regulation of Rab5a expression

The human Rab5a (NM_004162.4) 3^/^-flanking regulatory region was PCR amplified from total cDNA synthesized from THP-1 human macrophages using specific primers, Rab5a:Forward 5^/^-GTGAGCTCACCTCTAGTTTGAACTAGCTGG-3^/^ and Rab5a:Reverse 5^/^-GT CTCGAGGCTTTTTATACCACTTTATTCC-3^/^. The amplified product (1350 bp) was digested with *SacI/XhoI* and cloned downstream of the firefly luciferase coding sequence between *SacI* and *XhoI* sites of dual luciferase pmir-GLO reporter vector (Promega). Mutants in miR-494 target site of 3^/^-flanking regulatory region of human Rab5a was generated by PCR-mediated mutagenesis. First a mutated megaprimer was amplified using reported forward primer and mutated reverse primer (5^/^-CTCATTTTTCCCTATTGTCCGTGTCGTAGTGAGAGGGACC-3^/^) using WT construct as template. Subsequently, a second round of PCR was carried out using mutated megaprimer as a forward primer and Rab5a 3^/^-UTR specific reverse primer to generate mutated full length 3^/^-flanking regulatory fragment. The mutated fragment was also cloned into pmir-GLO vector as described.

To determine miR-494 mediated regulation of hereterologous expression of Rab5a chimeric construct, these chimeric reporter constructs were cotransfected with 40 nM miR-494 or control mimic miR into semiconfluent HeLa cells using Lipofectamine 2000 reagent (Invitrogen) as per manufacturer’s protocol. After 48 h, cells were lysed in 1X passive lysis buffer (Promega) and the lysates were used to measure Firefly and Renilla luciferase activity by Dual-Luciferase reporter assay kit (Promega). Renilla luciferase activity was used as a control reporter for normalization. Results were expressed as relative luciferase activity compared to miR-494 untreated control cell arbitrarily chosen as 1 unit.

#### Determination of miR-494 mediated expression of Rab5 isoforms

To determine miR-494 mediated regulation of endogenous expression of Rab5 isoforms, HeLa or THP-1 differentiated human macrophages were transfected with 40 nM miR-494 and levels of different isoforms of Rab5 were determined after 48 h by q PCR as described. Untransfected cells were used as control. Results were analyzed using comparative Ct method (2^-ΔΔCt^) and expressed as relative expression compared to untreated control arbitrarily chosen as 1 unit.

#### Immunofluorescence

For immunofluorescence studies, infected or uninfected cells were fixed in ice cold methanol for 10 min. Cells were washed three times with PBS and permeablized with PBS containing 0.1% Triton X-100 for 10 min at 24°C. Subsequently, cells were blocked with blocking buffer (PBS containing 3% BSA and 0.1% Triton X-100) for 2 h at RT. Permeabilized cells were further probed with specific antibodies against indicated protein in blocking buffer for 12 h at 4°C. Finally, cells were washed three times with PBS and incubated with Alexa Flour labeled secondary antibody (1:1,000) for 1 h at RT in blocking buffer. Cells were mounted in Prolong gold antifade mounting reagent and viewed in an LSM 510 Meta confocal microscope using an oil immersion 63X objective.

#### Overexpression of GFP-Rab5a and mutants in HeLa cells

GFP-Rab5a and its mutants were overexpressed in HeLa cells by Lipofectamine 2000 as per manufacturer’s protocol. Briefly, 0.25 × 10^6^ cells were seeded per well in a 6-well plate containing sterile coverslip and were allowed to grow for 12–16 h. Thereafter, cells were transfected with 1 μg of plasmid DNA and 2 μl Lipofectamine 2000 per ml of transfection mix per well in incomplete DMEM for 6 h. After 6 h, the transfection mix was replaced with complete DMEM and cells were allowed to grow for next 24 h. Thereafter, cells were fixed in 4% paraformaldehyde for 20 min at RT and then mounted in prolong gold antifade for 12 h at 4°C. Cells were visualized under confocal microscope.

#### Labeling of lysosomes

To label the lysosomes, THP-1 differentiated human macrophages were incubated with Fluoresbrite-YG-latex beads (2.00μm; Polysciences) with the multiplicity of 20 beads per macrophages for 3 h at 37°C. Cells were washed to remove uninternalized beads and incubated for additional 24 h at 37°C in complete RPMI medium. Subsequently, cells were fixed and immune-stained with lysosomal markers to label the lysosomal compartment.

In addition, cells were also incubated with 50 μg/ml of Dextran Texas Red (70,000 MW, molecular probes) for 3 h at 37°C, washed and chased for additional 24 h at 37°C to prelabel the lysosomes. Subsequently, internalization of Green fluorescent labelled latex beads were carried out in these cells as mentioned above. Finally, cells were mounted in Prolong gold antifade mounting reagent and viewed in an LSM 510 Meta confocal microscope using an oil immersion 63X objective.

To determine the proteolysis activity of the lysosomes of the *L*. *donovani* infected and uninfected human macrophages, cells were labeled with DQ-BSA Red which is a fluorogenic substrate for proteases [29]. Briefly, THP-1 differentiated macrophages were infected with *L*. *donovani* promastigotes (MOI 1:20) or latex beads as described previously. Subsequently, cells were incubated with 10 μg/ml of DQ-BSA Red for 1 h at 37°C. Cells were washed with fresh medium and incubated for another 3 h 37°C. Cells were mounted in Prolong gold antifade mounting reagent and viewed in an LSM 510 Meta confocal microscope using an oil immersion 63X objective. Total fluorescence per cell was determined using LSM 510 Meta software.

#### Determination of parasite load in human macrophages

THP-1 differentiated human macrophages were seeded on sterile glass coverslips placed in a 6-well plate. Subsequently, cells were transfected with 50 nM miR-494 or Rab5a specific siRNA using RNAi max transfection reagent (Invitrogen) as per manufacturer’s protocol and cells were incubated for 48 h at 37°C. Control RNA transfected cells were used as control. These cells were infected with *Leishmania* promastigotes as described earlier and chased for indicated periods of time. At respective time point, coverslips containing infected cells were washed three times in PBS and fixed with methanol for 10 min at 24°C. Coverslips were air dried and permeabilized with 0.4% saponin in PBS containing 0.1 mg/ml RNase A for 1 h at 37°C. Samples were washed and stained for 4 min with 50 mg/ml of PI, followed by three washes with PBS. Coverslips were mounted in ProLong gold antifade reagent (Molecular Probes) and viewed in a LSM510 confocal microscope (Zeiss) using an oil immersion 63X objective. Numbers of parasites present per macrophage were microscopically estimated and results are expressed as numbers of parasites present in 100 macrophages.

#### Statistical analysis

Statistical analysis was performed using Sigma Plot version 12. Student’s two-tailed paired t test or two tailed Mann-Whitney test was used to determine differences between control and test groups with 95% confidence intervals. P values less than 0.05 was considered to be significant for all analyses.

#### Ethical statement

All studies were performed following the guidelines provided by Institutional Bio-safety Committee of National Institute of Immunology, New Delhi, India (the approval number is IBSC #193/13). The animal care and experimental protocol for the animal experiments was approved by the Institutional Animal Ethics Committee of National Institute of Immunology, New Delhi, India (the approval number is IAEC# 377/15). Isolation of human PBMCs from healthy individuals after obtaining their consent was carried out by adhering to the approved guidelines of the Institutional Human Ethics Committee of National Institute of Immunology, New Delhi, India (the approval number is IHEC #96/17). The approval is as per the guidelines issued by Committee for the Purpose of Control and Supervision of Experiments on Animals (CPCSEA), Govt. of India.

## Supporting information

S1 Fig Determination of levels of mRNA of Rab5 isoforms in *Leishmania donovani* infected human macrophages by RT-PCR. To determine the levels of mRNA of Rab5 isoforms in *Leishmania* infected and uninfected human macrophages, total RNA was isolated using TRIzol reagent from respective cell lysate and used for cDNA synthesis. Briefly, PCR was performed with specific set of primers for the indicated Rabs using 2 μl of cDNA as template. PCR amplification was performed with initial denaturation for 5 min at 94°C followed by 28 cycles of amplification (denaturation for 30 sec at 94°C, annealing for 30 sec at 58°C and extension for 30 sec at 68°C) and final extension for 5 min at 68°C. 5 μl of amplified products were analyzed on 0.8% agarose gel. 18s rRNA amplification was used as loading control. All results are representative of three independent observations.
(TIF)

S2 Fig miRanda generated sequence alignment of human miR-494 binding site in the 3^/^-UTR of Rab5 isoforms. In order to investigate the specificity of miR-494 binding with Rab5 isoforms, miRanda–mirSVR algorithm (microRNA.org) was used. Based on the mirSVR scoring, the affinity of miR-494 binding to its complimentary site was highest for Rab5a (mirSVR score = −1.2494) as compared to Rab5b (mirSVR score = −0.0086) and Rab5c (mirSVR score: −0.1579).
(TIF)

S3 Fig Determination of the role of gp63 in the degradation of c-Jun in Raw 264.7 mouse macrophages. To directly determine the role of *Leishmania* gp63 in the degradation of c-Jun in infected macrophages, first the gp63 was cloned into p3XFLAG-Myc-CMV-26 vector. Subsequently, this construct was transfected into Raw 264.7 macrophages by electroporation. Transfection of vector alone was used as control. Cells were washed and further incubated for an additional 24 h at 37°C under similar conditions. Cells were lysed and level of c-Jun was determined from the vector transfected and gp63 overexpressed cells by Western blot analysis using specific antibody. Overexpression of gp63 was confirmed by Western blot analysis using anti-Flag antibody. Actin was used as controls. All results are representative of three independent observations.
(TIF)

S4 Fig Determination of the levels of Rab5a protein in miR-494 overexpressed HeLa cells. To determine the role of miR-494 in the expression of Rab5a, HeLa cells were transfected with 50 nM miR-494 or control mimic as described in Materials and Methods and level of Rab5a protein was determined after 48 h by Western blot analysis using anti-Rab5a antibody. Actin was used as a control. Results are represented as mean ± S.D. of three independent experiments and normalized to the actin control. Expression of Rab5a in control cells was arbitrarily chosen as one unit.
(TIF)

S5 Fig Determination of the specificity of the recruitment of Rab5a and EEA1 on *Leishmania*-PV in human macrophages. THP-1 differentiated macrophages were infected with *L*. *donoavni* and recruitment of Rab5 isoforms, EEA1, Rab9 and Rab8 on PV were determined after indicated time point of infection by immuno-staining with specific antibody as described in Materials and Methods. A. Recruitment of different isoforms of Rab5 on *Leishmania*-PV in human macrophages after 24 h of infection. B. Time dependent recruitment of Rab5a on *Leishmania*-PV in human macrophages. C. Time dependent recruitment of EEA1 on *Leishmania*-PV in human macrophages. D. Recruitment of Rab9 and Rab8 on *Leishmania*-PV in human macrophages after 24 h of infection. Cells were mounted in Prolong gold antifade mounting reagent and viewed in an LSM 510 Meta confocal microscope using an oil immersion 63X objective. All results are representative of three independent observations.
(TIF)

S6 Fig Determination of the expression and recruitment of Rab5a in *Leishmania donovani* infected Raw 264.7 mouse macrophages. Raw 264.7 mouse macrophages were infected with *L*. *donovani* promastigotes in RPMI medium at MOI of 1:20 for 3 h at 37°C as described in Materials and Methods for THP-1 differentiated human macrophages. A. To detect the levels of Rab5a in *Leishmania* infected and uninfected Raw 264.7 mouse macrophages, cells were lysed using lysis buffer for 60 min at 24°C and centrifuged at 15000 x g for 15 min. Subsequently, cellular proteins (40 μg) were resolved on a SDS–PAGE and Western blot analyses were carried out using anti-Rab5a antibody. Actin was used as control. B. Raw 264.7 mouse macrophages were infected with *L*. *donoavni* as described previously and recruitment of Rab5a was determined after indicated time point of infection by immuno-staining with anti-Rab5a as described in Materials and Methods. All results are representative of three independent observations.
(TIF)

S7 Fig Determination of localization of *Leishmania donovani* in human macrophages. *L*. *donovani* and latex beads were coinfected in THP-1 differentiated macrophages as described in Materials and Methods. After 24 h of infection, cells were permeabilized and probed with anti-Rab5a or anti-EEA1 antibody. Finally, cells were washed and stained with Alexa Flour labeled secondary antibody. Macrophage and *Leishmania* nucleus were stained with DRAQ5. Cells were mounted in Prolong gold antifade mounting reagent and viewed in an LSM 510 Meta confocal microscope using an oil immersion 63X objective. Red, Rab5a (upper panel) and EEA1 (Lower Panel); Green, Latex beads; Blue, Nucleus. All results are representative of three independent observations.
(TIF)

## Abbreviations

EE: Early Endosome
EEA1: Early Endosome Associated Antigen
Human macrophages: THP-1 differentiated human macrophage cell line
Lamp1: Lysosome Associated Membrane Protein 1
Ld: *Leishmania donovani*
miR: MicroRNA
PV: Parasitophorous Vacuole
