# Supplementary figures and images for: Leishmania donovani resides in modified early endosomes by upregulating Rab5a expression via the downregulation of miR-494
Source: PLoS Pathog. 2017 Jun 26;13(6):e1006459. doi: 10.1371/journal.ppat.1006459 (PMC5501680; doi:10.1371/journal.ppat.1006459)

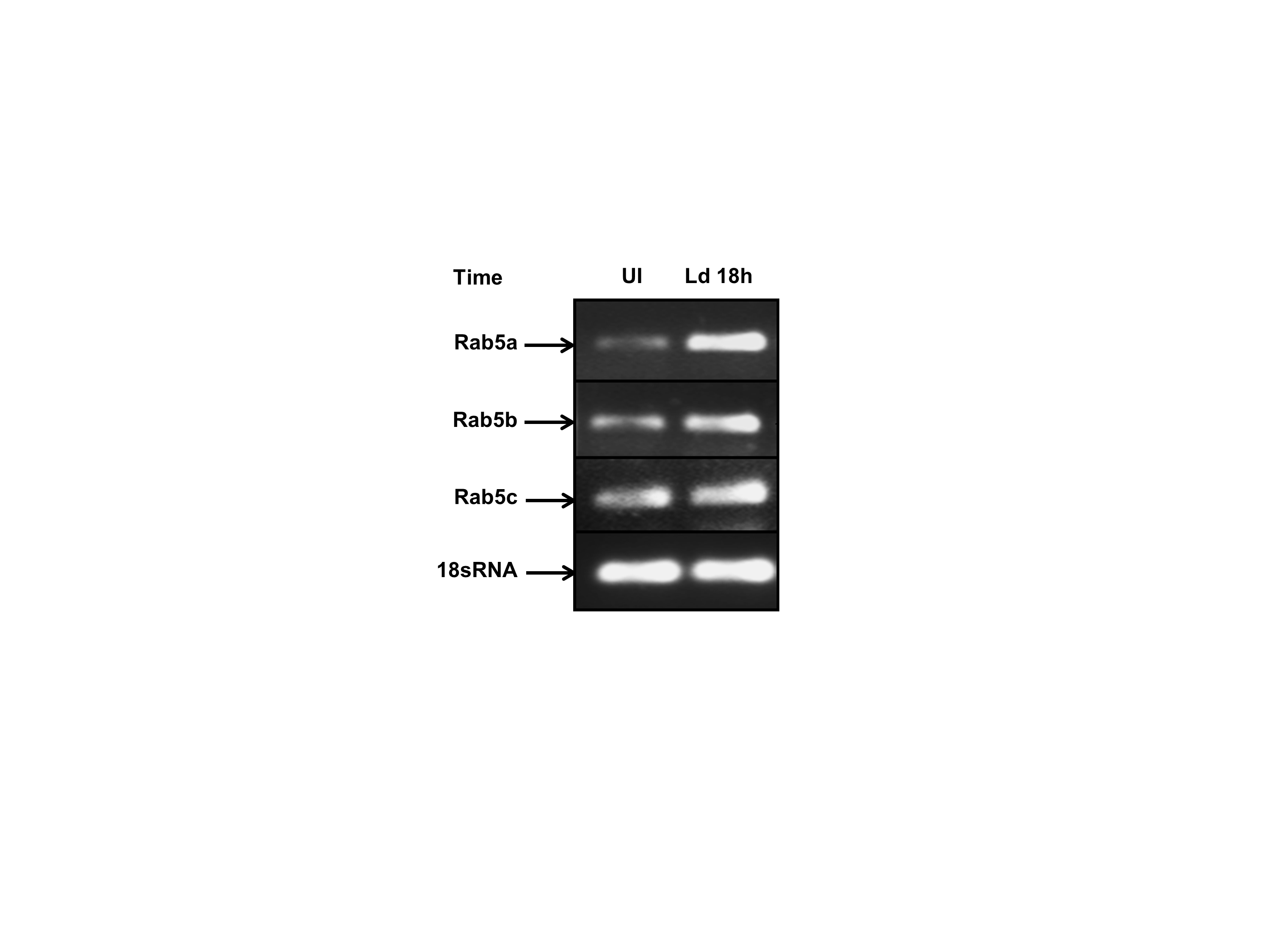

Supplement: S1 Fig — To determine the levels of mRNA of Rab5 isoforms in Leishmania infected and uninfected human macrophages, total RNA was isolated using TRIzol reagent from respective cell lysate and used for cDNA synthesis. Briefly, PCR was performed with specific set of primers for the indicated Rabs using 2 μl of cDNA as template. PCR amplification was performed with initial denaturation for 5 min at 94°C followed by 28 cycles of amplification (denaturation for 30 sec at 94°C, annealing for 30 sec at 58°C and extension for 30 sec at 68°C) and final extension for 5 min at 68°C. 5 μl of amplified products were analyzed on 0.8% agarose gel. 18s rRNA amplification was used as loading control. All results are representative of three independent observations. (TIF) [file ppat.1006459.s001.tif]

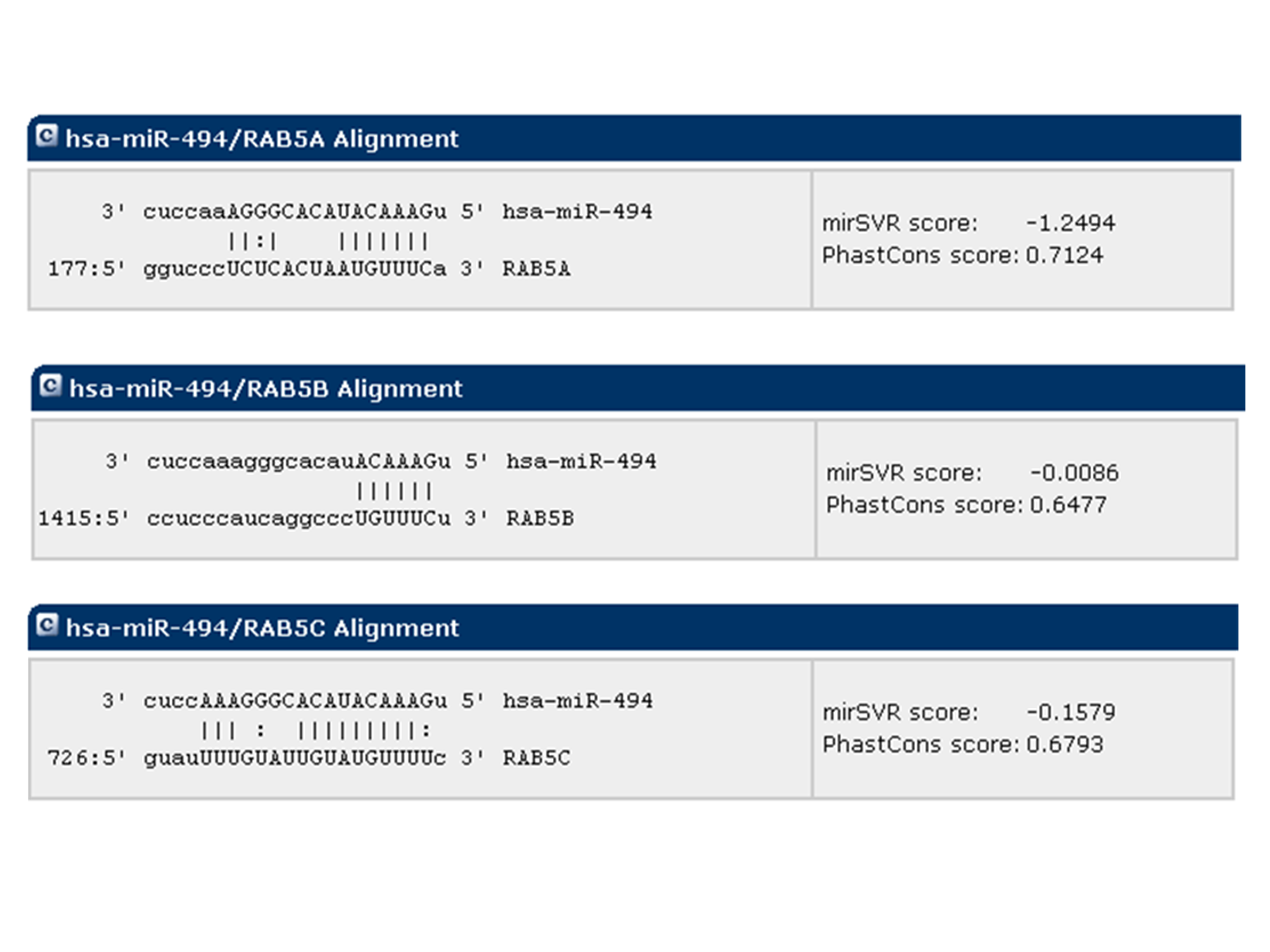

Supplement: S2 Fig — In order to investigate the specificity of miR-494 binding with Rab5 isoforms, miRanda–mirSVR algorithm (microRNA.org) was used. Based on the mirSVR scoring, the affinity of miR-494 binding to its complimentary site was highest for Rab5a (mirSVR score = −1.2494) as compared to Rab5b (mirSVR score = −0.0086) and Rab5c (mirSVR score: −0.1579). (TIF) [file ppat.1006459.s002.tif]

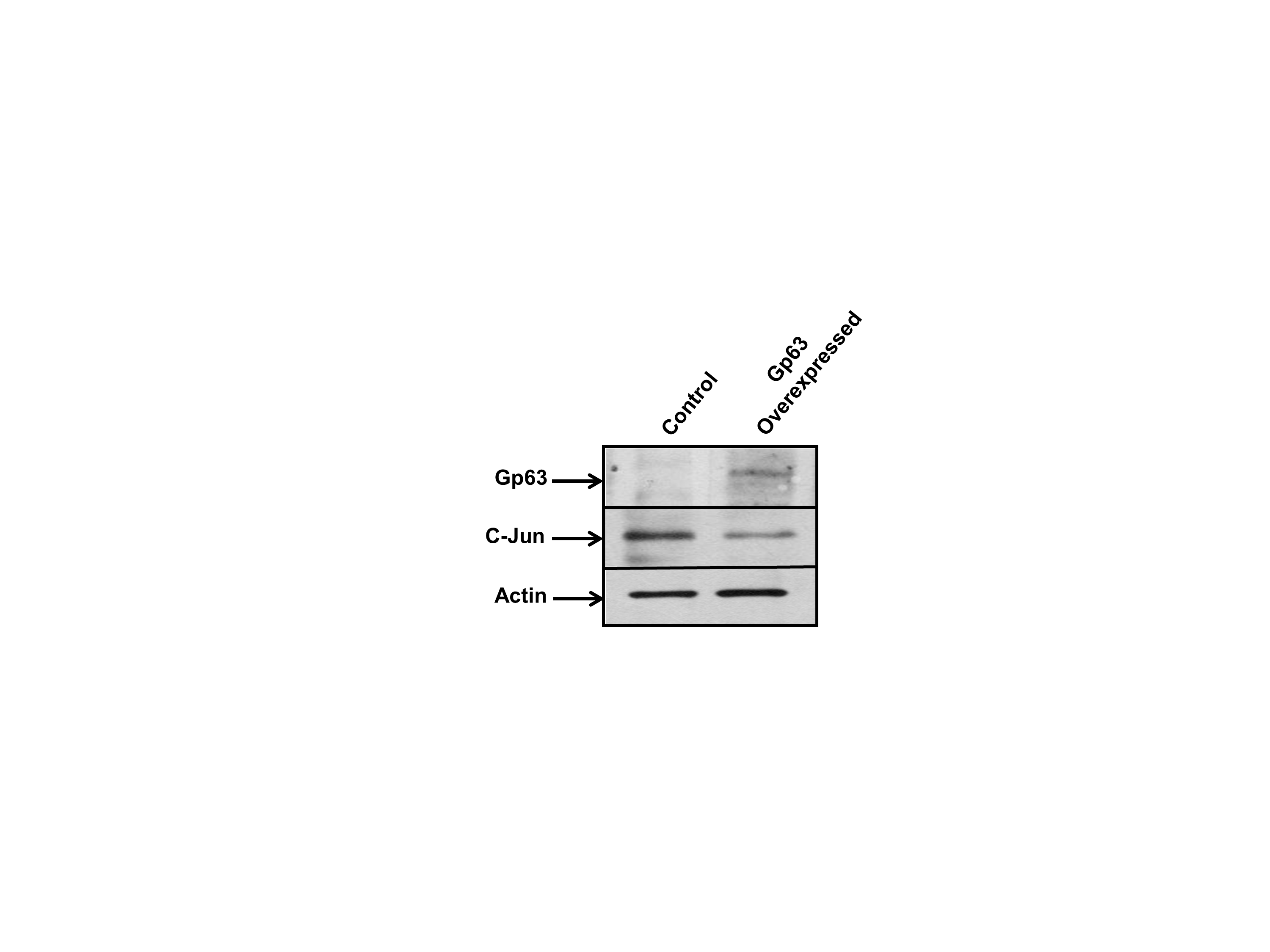

Supplement: S3 Fig — To directly determine the role of Leishmania gp63 in the degradation of c-Jun in infected macrophages, first the gp63 was cloned into p3XFLAG-Myc-CMV-26 vector. Subsequently, this construct was transfected into Raw 264.7 macrophages by electroporation. Transfection of vector alone was used as control. Cells were washed and further incubated for an additional 24 h at 37°C under similar conditions. Cells were lysed and level of c-Jun was determined from the vector transfected and gp63 overexpressed cells by Western blot analysis using specific antibody. Overexpression of gp63 was confirmed by Western blot analysis using anti-Flag antibody. Actin was used as controls. All results are representative of three independent observations. (TIF) [file ppat.1006459.s003.tif]

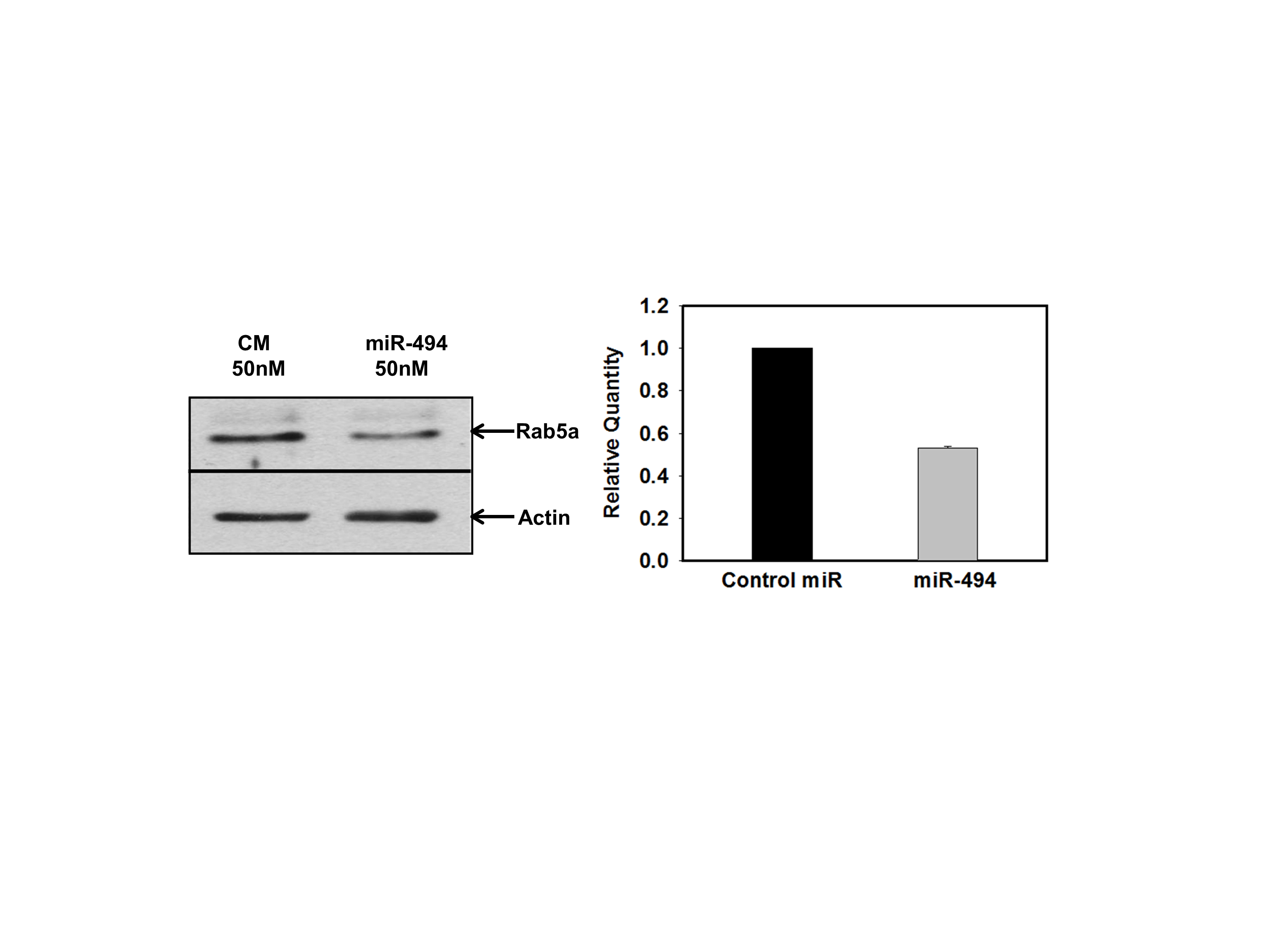

Supplement: S4 Fig — To determine the role of miR-494 in the expression of Rab5a, HeLa cells were transfected with 50 nM miR-494 or control mimic as described in Materials and Methods and level of Rab5a protein was determined after 48 h by Western blot analysis using anti-Rab5a antibody. Actin was used as a control. Results are represented as mean ± S.D. of three independent experiments and normalized to the actin control. Expression of Rab5a in control cells was arbitrarily chosen as one unit. (TIF) [file ppat.1006459.s004.tif]

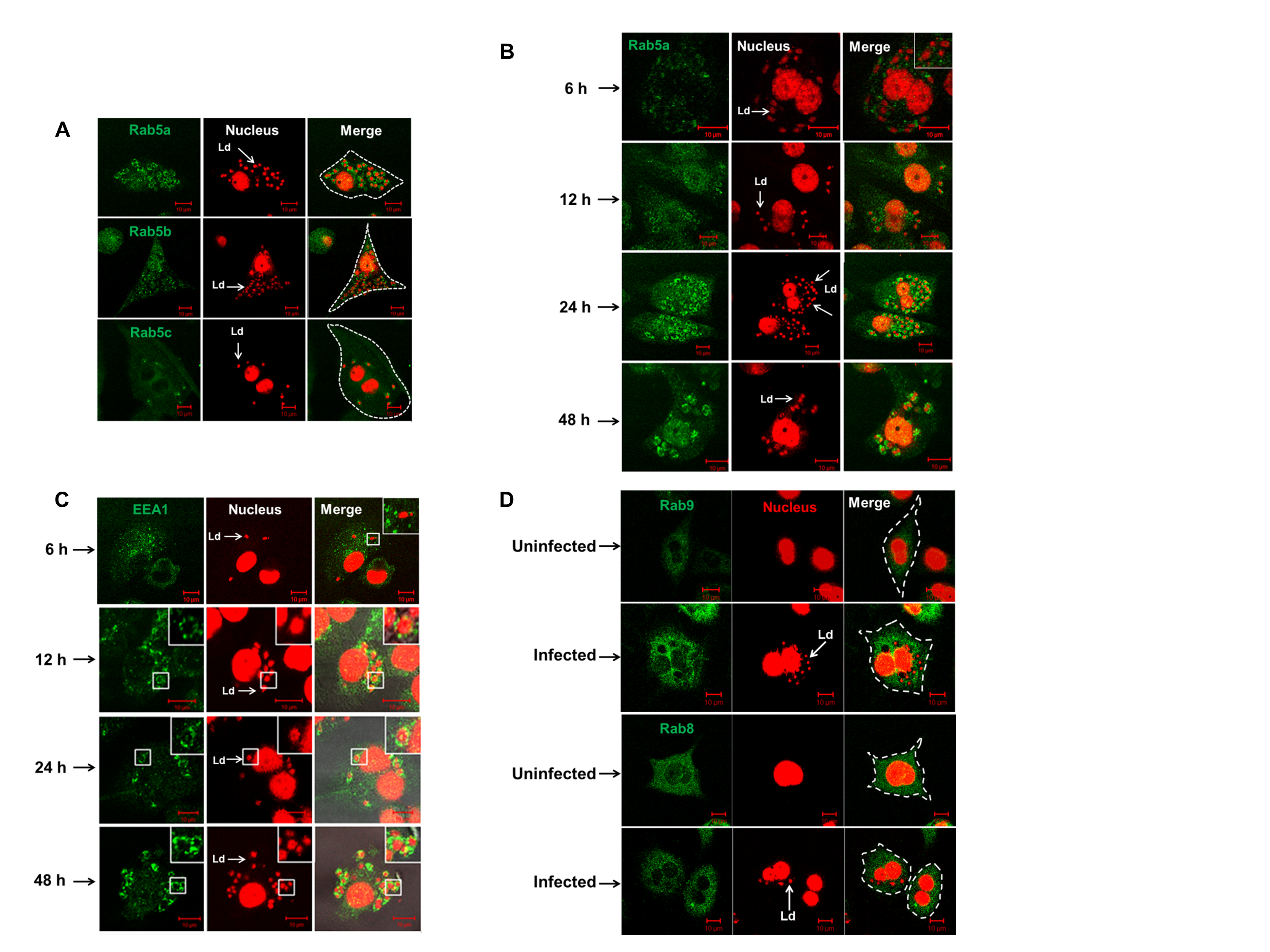

Supplement: S5 Fig — THP-1 differentiated macrophages were infected with L. donoavni and recruitment of Rab5 isoforms, EEA1, Rab9 and Rab8 on PV were determined after indicated time point of infection by immuno-staining with specific antibody as described in Materials and Methods. A. Recruitment of different isoforms of Rab5 on Leishmania-PV in human macrophages after 24 h of infection. B. Time dependent recruitment of Rab5a on Leishmania-PV in human macrophages. C. Time dependent recruitment of EEA1 on Leishmania-PV in human macrophages. D. Recruitment of Rab9 and Rab8 on Leishmania-PV in human macrophages after 24 h of infection. Cells were mounted in Prolong gold antifade mounting reagent and viewed in an LSM 510 Meta confocal microscope using an oil immersion 63X objective. All results are representative of three independent observations. (TIF) [file ppat.1006459.s005.tif]

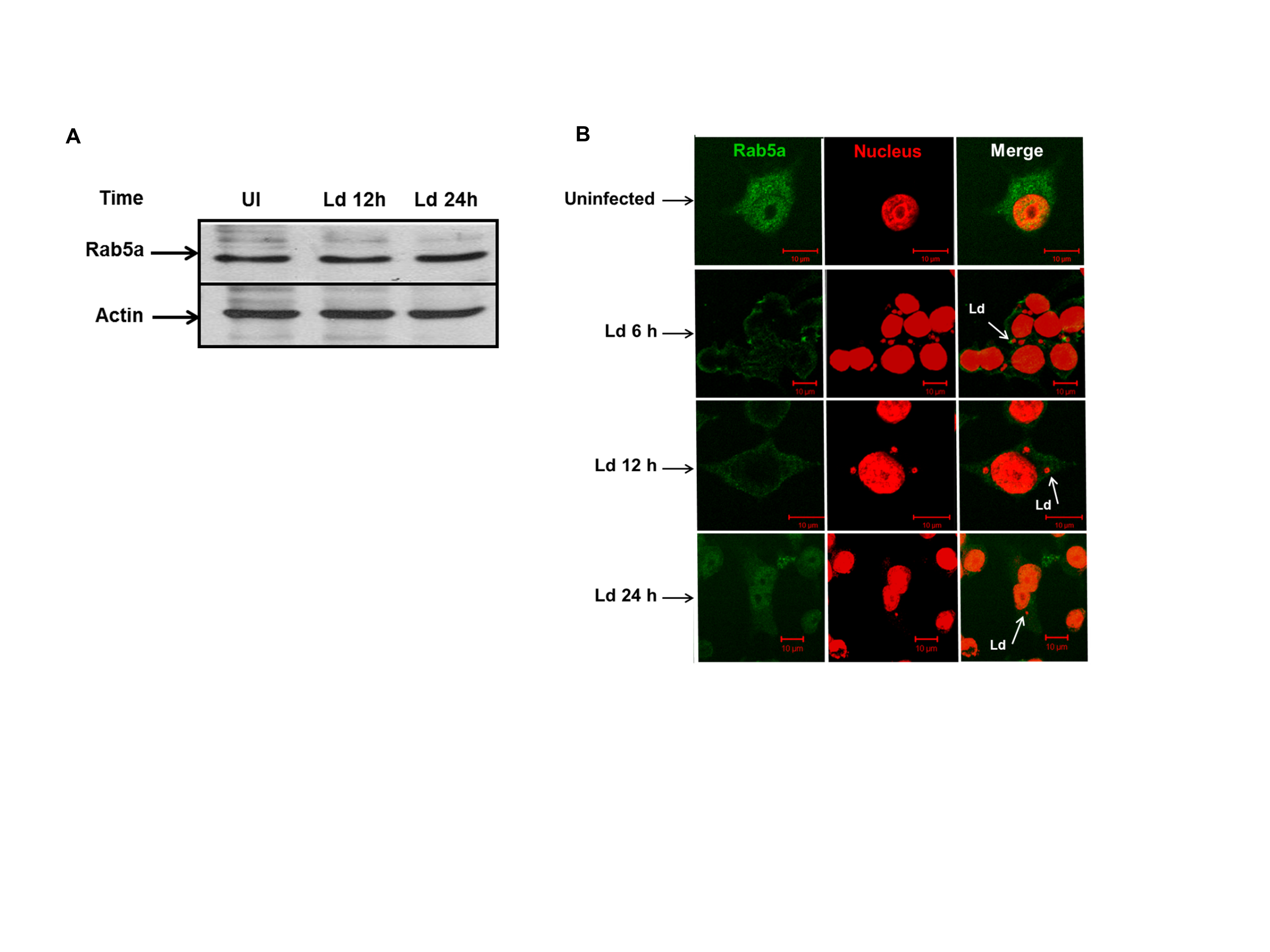

Supplement: S6 Fig — Raw 264.7 mouse macrophages were infected with L. donovani promastigotes in RPMI medium at MOI of 1:20 for 3 h at 37°C as described in Materials and Methods for THP-1 differentiated human macrophages. A. To detect the levels of Rab5a in Leishmania infected and uninfected Raw 264.7 mouse macrophages, cells were lysed using lysis buffer for 60 min at 24°C and centrifuged at 15000 x g for 15 min. Subsequently, cellular proteins (40 μg) were resolved on a SDS–PAGE and Western blot analyses were carried out using anti-Rab5a antibody. Actin was used as control. B. Raw 264.7 mouse macrophages were infected with L. donoavni as described previously and recruitment of Rab5a was determined after indicated time point of infection by immuno-staining with anti-Rab5a as described in Materials and Methods. All results are representative of three independent observations. (TIF) [file ppat.1006459.s006.tif]

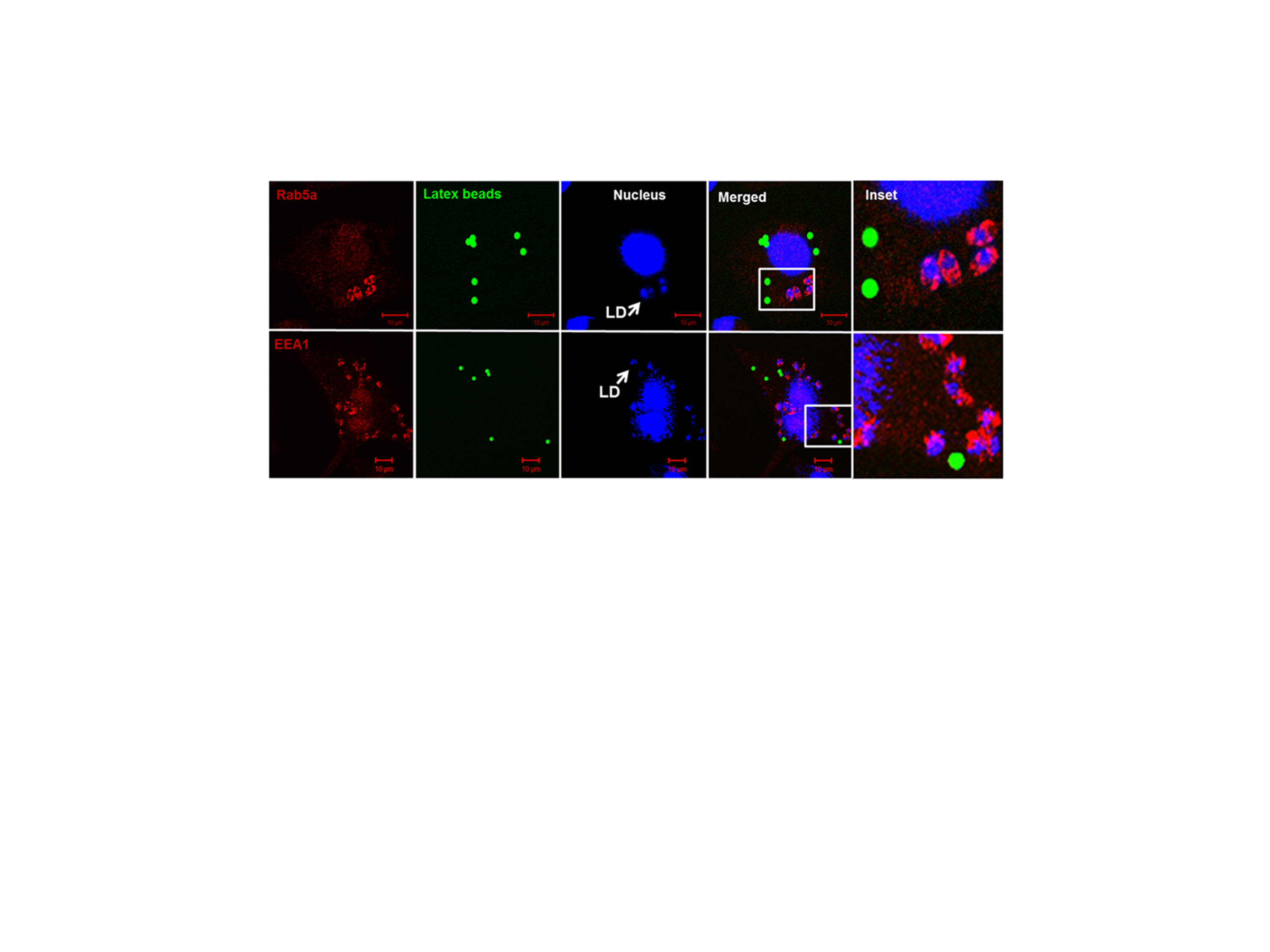

Supplement: S7 Fig — L. donovani and latex beads were coinfected in THP-1 differentiated macrophages as described in Materials and Methods. After 24 h of infection, cells were permeabilized and probed with anti-Rab5a or anti-EEA1 antibody. Finally, cells were washed and stained with Alexa Flour labeled secondary antibody. Macrophage and Leishmania nucleus were stained with DRAQ5. Cells were mounted in Prolong gold antifade mounting reagent and viewed in an LSM 510 Meta confocal microscope using an oil immersion 63X objective. Red, Rab5a (upper panel) and EEA1 (Lower Panel); Green, Latex beads; Blue, Nucleus. All results are representative of three independent observations. (TIF) [file ppat.1006459.s007.tif]
